# Timing of renal replacement therapy initiation for acute kidney injury in critically ill patients: a systematic review of randomized clinical trials with meta-analysis and trial sequential analysis

**DOI:** 10.1186/s13054-020-03451-y

**Published:** 2021-01-06

**Authors:** Xiaoming Li, Chao Liu, Zhi Mao, Qinglin Li, Feihu Zhou

**Affiliations:** 1 Department of Critical Care Medicine, the First Medical Centre, Chinese People’s Liberation Army General Hospital, 28 Fu-Xing Road, Beijing, 100853 People’s Republic of China; 2 Medical School of Chinese PLA, Beijing, People’s Republic of China

**Keywords:** Renal replacement therapy, Acute kidney injury, Critically ill, Time, Systematic review, Meta-analysis

## Abstract

**Background:**

Acute kidney injury (AKI) is a common serious complication in critically ill patients. AKI occurs in up to 50% patients in intensive care unit (ICU), with poor clinical prognosis. Renal replacement therapy (RRT) has been widely used in critically ill patients with AKI. However, in patients without urgent indications such as acute pulmonary edema, severe acidosis, and severe hyperkalemia, the optimal timing of RRT initiation is still under debate. We conducted this systematic review of randomized clinical trials (RCTs) with meta-analysis and trial sequential analysis (TSA) to compare the effects of early RRT initiation versus delayed RRT initiation.

**Methods:**

We searched databases (PubMed, EMBASE and Cochrane Library) from inception through to July 20, 2020, to identify eligible RCTs. The primary outcome was 28-day mortality. Two authors extracted the data independently. When the *I*^2^ values < 25%, we used fixed-effect mode. Otherwise, the random effects model was used as appropriate. TSA was performed to control the risk of random errors and assess whether the results in our meta-analysis were conclusive.

**Results:**

Eleven studies involving 5086 patients were identified. Two studies included patients with sepsis, one study included patients with shock after cardiac surgery, and eight others included mixed populations. The criteria for the initiation of RRT, the definition of AKI, and RRT modalities existed great variations among the studies. The median time of RRT initiation across studies ranged from 2 to 7.6 h in the early RRT group and 21 to 57 h in the delayed RRT group. The pooled results showed that early initiation of RRT could not decrease 28-day all-cause mortality compared with delayed RRT (RR 1.01; 95% CI 0.94–1.09; *P* = 0.77; *I*^2^ = 0%). TSA result showed that the required information size was 2949. The cumulative *Z* curve crossed the futility boundary and reached the required information size. In addition, early initiation of RRT could lead to unnecessary RRT exposure in some patients and was associated with a higher incidence of hypotension (RR 1.42; 95% CI 1.23–1.63; *P* < 0.00001; *I*^2^ = 8%) and RRT-associated infection events (RR 1.34; 95% CI 1.01–1.78; *P* = 0.04; *I*^2^ = 0%).

**Conclusions:**

This meta-analysis suggested that early initiation of RRT was not associated with survival benefit in critically ill patients with AKI. In addition, early initiation of RRT could lead to unnecessary RRT exposure in some patients, resulting in a waste of health resources and a higher incidence of RRT-associated adverse events. Maybe, only critically ill patients with a clear and hard indication, such as severe acidosis, pulmonary edema, and hyperkalemia, could benefit from early initiation of RRT.

## Introduction

Acute kidney injury (AKI) is a common serious complication of critically ill patients. AKI occurs in up to 50% patients in intensive care unit (ICU), with poor clinical prognosis [1–4]. Patients with AKI are characteristic of a rapid loss of the kidney function, which can lead to electrolyte disorder, metabolic acidosis, fluid overload, and an increase in serum uremic toxins. Renal replacement therapy (RRT) has been widely used in critically ill patients with AKI. For patients with severe complications such as acute pulmonary edema, severe acidosis, and severe hyperkalemia, RRT is the cornerstone of AKI treatment to be performed urgently [5, 6]. However, without these urgent indications, the optimal timing of initiating RRT is still under debate. Early initiation of RRT can correct metabolic disorders, control disturbances of fluid metabolism, and remove uremic toxins quickly and effectively. However, for patients whose renal function can recover spontaneously, early initiation of RRT may not be beneficial but expose them to the risk of RRT-associated adverse events, such as hemodynamic instability, bleeding, and bloodstream infection [6–8].

Although there were several meta-analyses to evaluate whether critically ill patients with AKI can benefit from initiating RRT early, the conclusions were inconsistent and none of them included all randomized clinical trials (RCTs) up to present. Karvellas et al. conducted a meta-analysis, including two RCTs, four prospective cohort, and nine retrospective cohort, showing a beneficial impact on survival when RRT was performed at early stage [9]. However, recently published meta-analyses on this topic indicated that early initiation of RRT did not improve patient prognosis [10–12]. And a high-quality meta-analysis of RCTs with individual data of all the included patients reached the similar conclusion [13]. Recently, the largest RCT, STARRT‑AKI trail, was published. Totally, 2927 critically ill patients with severe AKI were randomly assigned to accelerated-strategy group and standard-strategy group. The primary and secondary outcomes were comparable between the two groups, while more adverse events occurred in the accelerated-strategy group [14].

Based on a sufficient number of high-quality RCTs, we conducted this systematic review of RCTs with meta-analysis and trial sequential analysis (TSA) to compare the effects of early RRT initiation versus delayed RRT initiation.

## Methods

We followed the Preferred Reporting Items for Systematic Reviews and Meta-Analyses (PRISMA statement) guidelines to perform this meta-analysis [15] (see Additional file 1). No prospectively registered protocol was existed; however, search terms, data extraction, inclusion and exclusion criteria, and data synthesis were according to a plan made by our team.

### Eligibility criteria

The inclusion criteria were as follows: (1) population: critically ill patients with AKI aged 18 years or older; (2) intervention: the treatment group received early RRT; (3) Comparison intervention: the control group received delayed RRT; (4) outcome: 28-day all-cause mortality, 90-day mortality, or hospital all-cause mortality were available; and (5) study design: RCT. The exclusion criteria were as follows: (1) study type was not RCT; (2) patients included children; (3) study not focused on critical illness; (4) without a clearly definition of “early” and “delayed” strategies; and (5) the reason for initiating RRT was not AKI, but others. There were no restrictions on publication language.

### Search strategy and selection process

We searched PubMed, EMBASE, and the Cochrane Central Register of Controlled Trials Library database from inception through to July 20, 2020. We used key-words and free-text words which were related to AKI, RRT, critical illness and timing of initiating RRT. The detail of search strategy for PubMed is shown in Additional file 2. The reference lists of the included studies and recent review articles were hand-searched to find additional citations. Two authors (X.L and C.L) independently screened all potentially relevant citations to find studies for the final analysis. Any disagreements between two authors were resolved through discussion.

### Data extraction and risk of bias assessment

Two authors (X.L and C.L) extracted the following information in a standard form independently: the first author, study center (single-center or multicenter trial), publication year, patient characteristics (mean age of the patient, sample size, male percentage and patient population), details of RRT (criteria for RRT initiation and RRT modality), all clinical outcomes. Two authors (X.L and C.L) independently evaluated the risk of bias for each of these studies by the Cochrane risk of bias assessment tool [16]. Any disagreements were resolved by discussion, if no agreement could be reached, it would be decided by a third author (F.Z). Only when all the items were assessed as low risk bias, the study was classified as low risk of bias, otherwise the study would be considered as high risk of bias.

### Outcomes

The primary outcome was 28-day all-cause mortality. The secondary outcomes included 90-day all-cause mortality, hospital all-cause mortality, ICU all-cause mortality, number of patients who received RRT, RRT dependence at 28-day among survivors, RRT dependence at 90-day among survivors, length of hospital stay, length of ICU stay, mechanical ventilation-free days up to day 28, RRT-free days up to day 28, and vasopressor-free days up to day 28. The incidence of adverse events potentially associated with RRT was also evaluated, including hypotension, any arrhythmia, bleeding events, and infection during the treatment.

### Statistical analysis

For binary outcomes, we calculated the risk ratios (RRs) and 95% confidence intervals (CIs) by the Mantel–Haenszel method. For continuous outcomes, we used the inverse variance method to pool the mean differences (MDs) and 95% CIs. Heterogeneity among the included studies was assessed using the *I*^2^ statistic, which the *I*^2^ values of 25%, 50%, and 75% represented low, moderate, and high heterogeneity, respectively [17]. When the *I*^2^ values < 25%, we used the fixed-effect mode. Otherwise, the random effects model was used as appropriate. If a two-sided P value was less than 0.05, the results were considered statistically significant. We used funnel plots to assess the publication bias [18]. Subgroup analyses for the primary outcome were performed based on mean age of patients in each study (> 65 years or ≤ 65 years), the SOFA scores at administration (> 12 or ≤ 12), and the criteria for early RRT initiation (Approximately equal to stage 2 of the KDIGO classification, approximately equal to stage 3 of the KDIGO classification, or other classification criteria subgroup) [19]. We did sensitivity analyses for the primary outcome according to publish language (excluding the study published in Chinese), risk of bias (only including studies classified as low risk of bias), and publish year (removing studies published before 2010). All statistical analyses were performed by Review Manager (version 5.3).

### Trial sequential analysis

We conducted TSA to control the risk of random errors and assess whether the results in our meta-analysis were conclusive. We used a random effects model to construct the cumulative *Z* curve. TSA was performed to maintain an overall 5% risk of a type I error. Based on previous high-quality RCTs on this topic [14, 20], we used an anticipated relative risk reduction (RRR) of 15.0% with a power of 90% to calculate the required information size to detect or reject an intervention effect. And the control event rate was adjusted according to the relevant rate of standard therapy (delayed-strategy) group in our meta-analysis. When the cumulative *Z *curve crossed the trial sequential monitoring boundary or entered the futility area, a sufficient level of evidence for accepting or rejecting the anticipated intervention effect may have been reached, and no further studies were needed. If the *Z *curve did not cross any of the boundaries, and the required information size had not been reached, evidence to reach a conclusion was insufficient, and more studies would be required [21].

## Results

### Selection of included studies

**Fig. 1:**
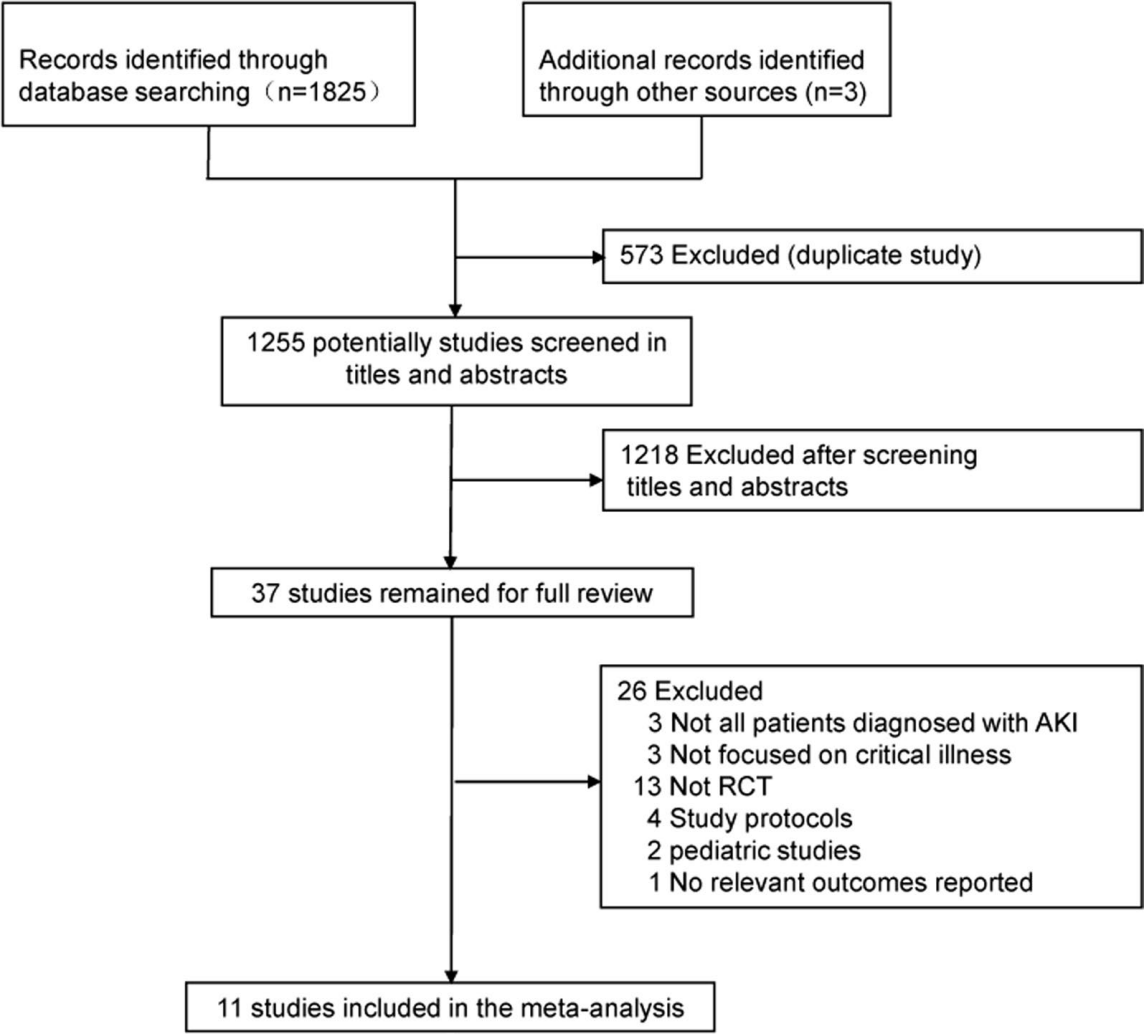
Flow diagram for the identification of eligible studies

According to our search strategy, 1828 potentially studies were identified. Five hundred seventy-three duplicate publications were excluded. Thirty-seven studies were eligible for full-text reviews after screening titles and abstracts. Only eleven studies involving 5086 patients were finally included in this meta-analysis [14, 20, 22–30] (Fig. 1). Table 1 summarizes the characteristics of the included individual studies. Most studies were assessed as low risk of bias [14, 20, 22–27] (see Additional file 3). Eight studies were done in a multicenter, while three studies were done in a single-center. All other studies were published after 2010, except for one study. The main cause of AKI in the included studies was sepsis. The number of participants across studies ranged from 40 to 2927. The range of the mean age of study participants was 42.4–69. The criteria for early initiation of RRT and delayed initiation of RRT existed differences among the studies. Due to the different criteria of initiating RRT, the time of initiating RRT was different. The median time of RRT initiation across studies ranged from 2 to 7.6 h in the early RRT group and 21 to 57 h in the delayed RRT group.

**Table 1:** Characteristics of included studies

Study	Country	Design and setting	Total no. of patients (male)/mean age (SD)	Criteria for RRT initiation		RRT modality	Median time of RRT initiation (IQR), h		SOFA scores at administration
				Early	Delayed		Early	Delayed	
Bagshaw [14]	Canada	Multicenter Mixed population	2927 (1990) 64.6 + 14.9	within 12 h after the onset of KDIGO stage 2 or 3	Serum potassium ≥ 6 mmol/; pH ≤ 7.2; serum bicarbonate ≤ 12 mmol/L; severe respiratory failure; volume overload; persistent AKI ≥ 72 h	IHD, CRRT	6.1 [3.9,8.8]	31.1 [19.0,71.8]	11.7 + 3.6
Barbar [22]	France	Multicenter Septic shock population	488 (296) 69 + 12.2	Within 12 h after the onset of failure stage of RIFLE	Serum potassium ≥ 6.5 mmol/; pH ≤ 7.15; severe pulmonary edema refractory to diuretics; no renal function recovery after 48 h	IHD, CRRT	7.6 [4.4,11.5]	51.5 [34.6,59.5]	12.3 + 2.9
Bouman [29]	Netherlands	Two-center Mixed population	71 (42) 68.5 + 11.6	Within 12 h if urine output < 30 ml/h for 6 h; creatinine clearance of < 20 mL/min	Plasma urea level of > 40 mmol/L; potassium of > 6.5 mmol/L or severe pulmonary edema	LVHF	7 [5, 10]	41.8 [21.4,72]	10.4 ± 2.1
Combes [23]	France	Multicenter Postcardiac surgery shock	224 (177) 59.5 ± 15.1	Persistent postoperative shock after cardiac surgery	AKI Network stage 3; serum urea > 36 mmol/L; life-threatening hyperkalemia	HVHF, CVVHDF, IHD	NA	NA	11.8 ± 2.9
Gaudry [20]	France	Multicenter Mixed population	619 (409) 66.1 ± 13.9	KDIGO stage 3	Serum potassium > 6 mmol/L; severe pulmonary edema refractory to diuretics; pH < 7.15; serum urea > 40 mmol/L; oliguria > 72 h	IHD, CRRT	2 [1, 3]	57 [25,83]	10.9 ± 3.1
Jamale [28]	India	Single-center Mixed population	208 (141) 42.4 ± 15.3	Serum urea nitrogen level > 70 mg/dL; creatinine level > 7 mg/dL	Refractory hyperkalemia; volume overload; and acidosis; uremic nausea and anorexia	IHD	NA	NA	7.9 ± 3.2
Lumlertgul [25]	Thailand	Multicenter Mixed population	118 (58) 67.1 ± 15.8	AKI (any stage of KDIGO) and no response to furosemide stress test	BUN ≥ 100 mg/dL; serum potassium > 6 mmol/L; serum bicarbonate < 12 mmol/L or pH < 7.15; PaO2/FiO2 ratio < 200; severe pulmonary edema	CVVH, PIRRT, IHD	2 [1, 3]	21 [17,49]	12 ± 3.7
Srisawat [26]	Thailand	Two-center Mixed population	40 (22) 66.8 ± 15.9	AKI (any stage of RIFLE)	potassium > 6.2 mmol/L; pH < 7.2, severe pulmonary edema refractory to diuretics; persistent oliguria or anuria; serum urea > 40 mg/dL	CRRT	NA	NA	9.3 ± 3.6
Wald [27]	Canada	Multicenter Mixed population	100 (72) 63.1 ± 12.8	At least two of the following: twofold increase in serum creatinine from baseline; urine output < 6 mL/kg in the preceding 12 h; whole-blood NGAL ≥ 400 ng/m	Serum potassium > 6.2 mmol/L; serum bicarbonate < 10 mmol/L, severe pulmonary edema; persistent AKI for > 72 h	IHD, CRRT, SLED	7.4 [6.1,9.6]	31.6 [22.8,59.5]	13 ± 2.8
Xia [30]	China	Single-center Sepsis population	60 (33) 66.4 ± 11.5	Sepsis and urinary NGAL ≥ 1310 ng/mL	Serum potassium > 6.5 mmol/L; pH < 7.2; severe pulmonary edema	CRRT	NA	NA	9.6 ± 3.2
Zarbock [24]	Germany	Single-center Mixed population	231 (146) 67 ± 13.1	Within 8 h of diagnosis of KDIGO stage 2	Within 12 h of diagnosis of KDIGO stage 3; or serum urea > 100 mg/dL; serum potassium > 6 mmol/L; serum magnesium > 4 mmol/L; urine < 200 mL per 12 h or anuria; organ edema refractory to diuretics	CRRT, SLED, IHD	6 [4, 7]	25.5 [18.8,40.3]	15.8 ± 2.3

*AKI *acute kidney injury, *KDIGO *Kidney Disease Improving Global Outcomes classification, *RIFLE *risk, injury, failure, loss, and end-stage kidney disease classification system, *IHD *intermittent hemodialysis, *CRRT *continuous renal replacement therapy, *LVHF *low-volume hemofiltration, *HVHF *high-volume hemofiltration, *CVVHDF *continuous venovenous hemodiafiltration, *BUN *blood urea nitrogen, *CVVH *continuous venovenous hemofiltration, *PIRRT *prolonged intermittent renal replacement therapy, *SLED *sustained low-efficiency dialysis, *NA *not available, *SD* standard deviation

### Mortality

**Fig. 2:**
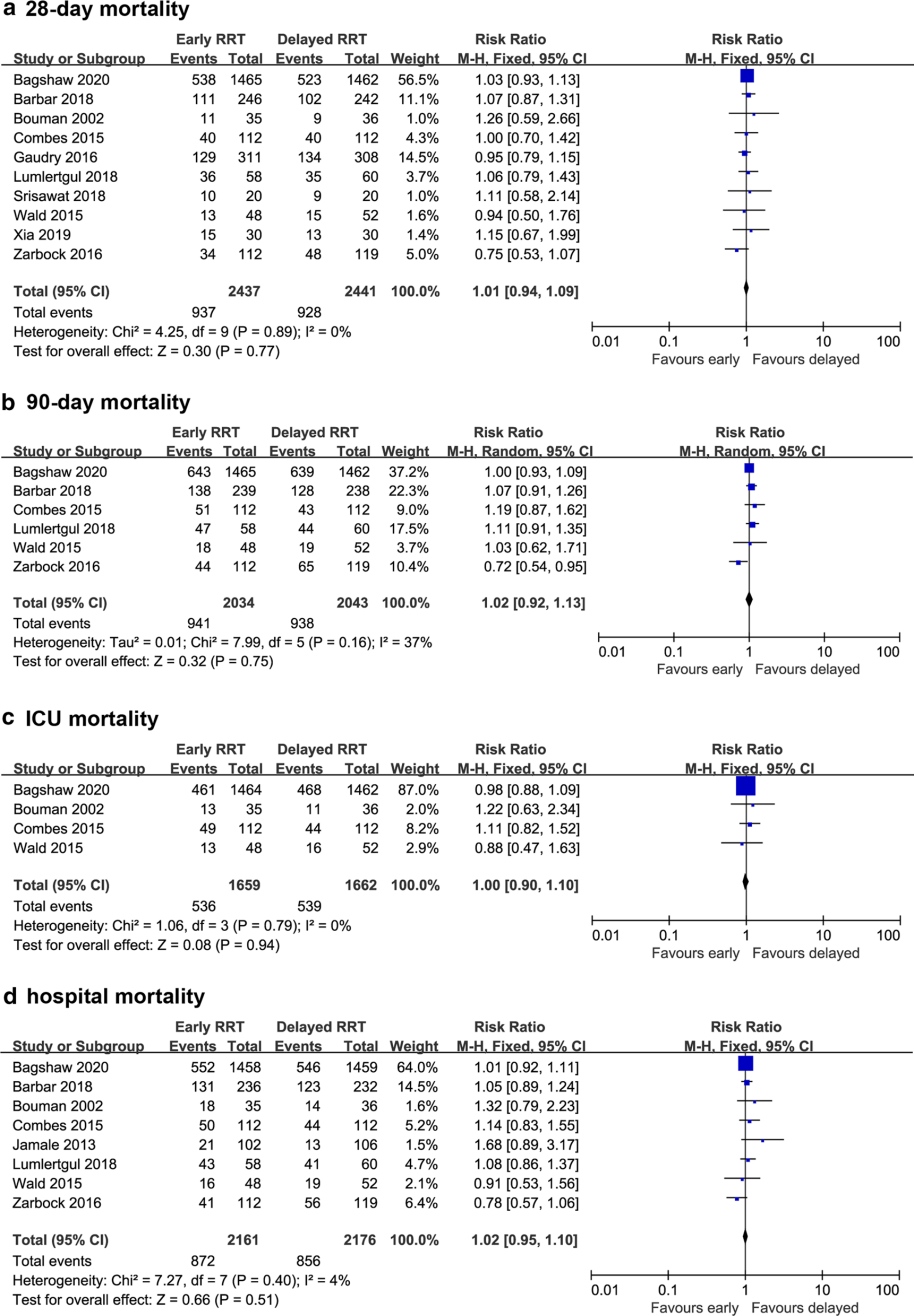
Forest plot of comparison: early RRT initiation group versus delayed RRT initiation group. **a** 28-day mortality; **b** 90-day mortality; **c** ICU mortality; d. hospital mortality

**Fig. 3:**
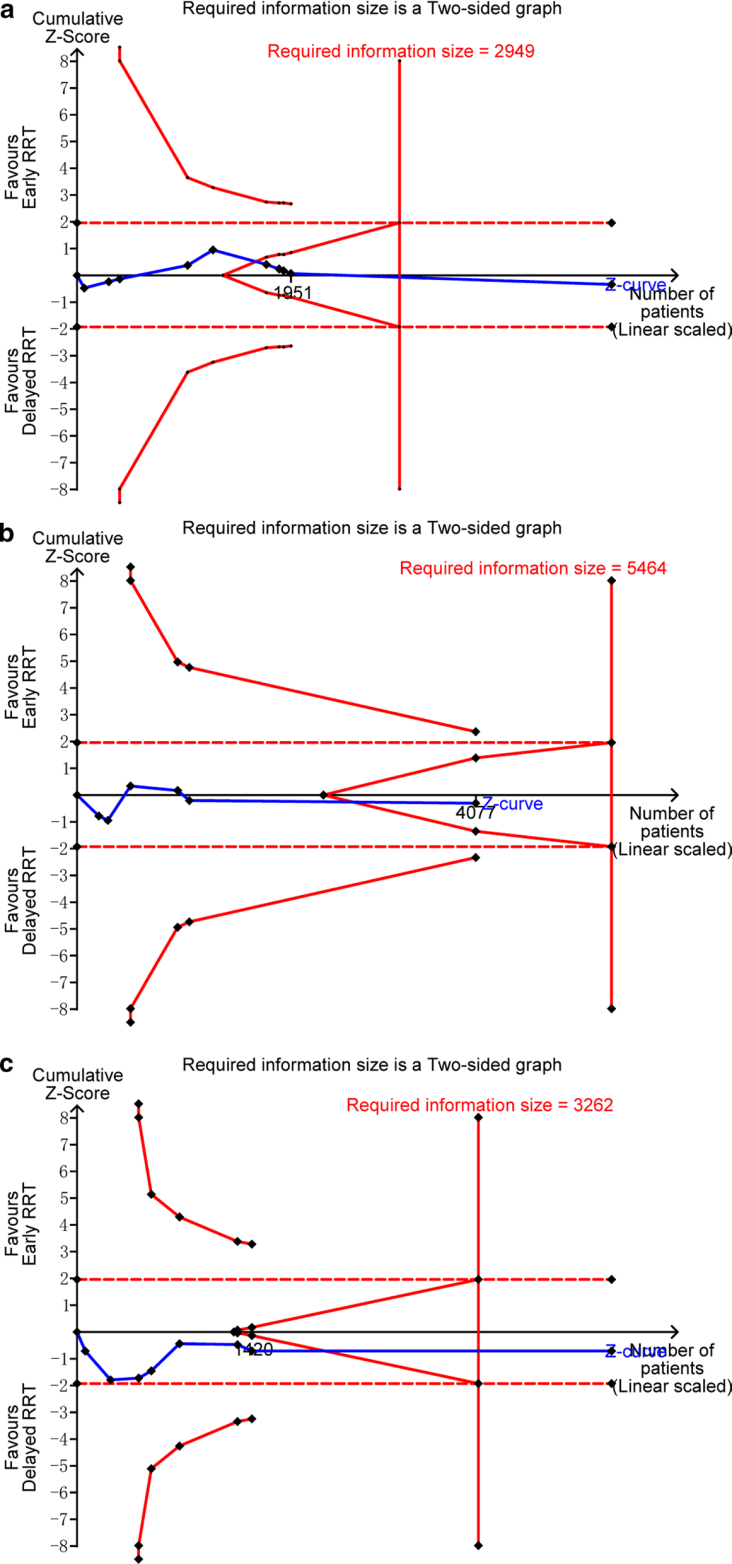
Trial sequential analysis. **a–c** The cumulative *Z* curve (complete blue line) was constructed using a random effects model. Etched red line shows conventional test boundary. Complete red line represents the trial sequential monitoring boundary. **a.** TSA for 28-day mortality. A diversity-adjusted information size of 2949 patients was calculated on the basis of using *α* = 0.05 (two sided), *β* = 0.10 (power 90%), an anticipated relative risk reduction (RRR) of 15.0%, and a control event rate of 38.0%. The cumulative *Z* curve crossed the futility boundary and reached the required information size. **b** TSA for 90-day mortality. A diversity-adjusted information size of 5464 patients was calculated on the basis of using *α* = 0.05 (two sided), *β* = 0.10 (power 90%), an anticipated relative risk reduction (RRR) of 15.0%, and a control event rate of 45.9%. The cumulative *Z* curve crossed the futility boundary. **c.** TSA for hospital mortality. A diversity-adjusted information size of 3262 patients was calculated on the basis of using *α* = 0.05 (two sided), *β* = 0.10 (power 90%), an anticipated relative risk reduction (RRR) of 15.0%, and a control event rate of 39.3%. The cumulative *Z* curve crossed the futility boundary and reached the required information size

Ten studies reported 28-day mortality [14, 20, 22–27, 29, 30]. The mortality in the early-strategy group and the delayed-strategy group was 38.4% (937 of 2437 patients) and 38.0% (928 of 2441 patients), respectively. The pooled results showed that early initiation of RRT could not decrease 28-day all-cause mortality compared with delayed initiation of RRT (RR 1.01; 95% CI 0.94–1.09; *P* = 0.77; *I*^2^ = 0%; Fig. 2a). There was no obvious asymmetry in funnel plots by visually inspecting (see Additional file 4). The TSA result showed that the required information size was 2949. The cumulative *Z* curve crossed the futility boundary and reached the required information size, suggesting that a RRR of 15% or greater could be rejected (Fig. 3a).

There were no significant differences in 90-day mortality (RR 1.02; 95% CI 0.92–1.13; *P* = 0.75; *I*^2^ = 37%; 4077 participants, 6 studies, Fig. 2b), ICU mortality (RR 1.00; 95% CI 0.90–1.10; *P* = 0.94; *I*^2^ = 0%; 3321 participants, 4 studies, Fig. 2c), or hospital mortality (RR 1.02; 95% CI 0.95–1.10; *P* = 0.51; *I*^2^ = 4%; 4337 participants, 8 studies, Fig. 2d) between the two groups. For 90-day mortality, TSA indicated that only 74.6% (4077 of 5464 patients) of the required information size was accrued. The cumulative *Z* curve did not cross the conventional boundary or the sequential monitoring boundary. However, the cumulative *Z* curve crossed the futility boundary (Fig. 3b). In terms of hospital mortality, the cumulative *Z* curve crossed the futility boundary and reached the required information size (Fig. 3c).

### Number of patients who received RRT

97.2% (2468 of 2539) of patients in the early-strategy group and 62.5% (1591 of 2547) of patients in the delayed-strategy group received RRT during therapy. Delayed initiation of RRT could significantly reduce the number of patients receiving RRT (RR 1.52; 95% CI 1.30–1.78; *P* < 0.00001; *I*^2^ = 95%; see Additional file 5a), indicating that renal function can recover spontaneously in a considerable proportion of AKI patients.

### RRT dependence among survivors

Six studies [20, 22–24, 26, 29] with 984 patients reported the number of patients who required RRT among survivors at 28 days, and five studies [14, 22–24, 27] with 2153 patients reported the number of patients who required RRT among survivors at 90 days. For survivors requiring RRT, no significant differences were detected between the two groups at 28 days (RR 0.97; 95% CI 0.58–1.60; *P* = 0.89; *I*^2^ = 46%; see Additional file 5b) and 90 days (RR 1.24; 95% CI 0.70–2.21; *P* = 0.46; *I*^2^ = 30%; see Additional file 5c), indicating that timing of RRT initiation was not associated with renal function recovery.

### Length of ICU stay and hospital stay

Data on the length of ICU stay and hospital stay were available in five studies [22, 24, 25, 27, 29] and seven studies [20, 22, 24, 25, 27–29], respectively. The pooled results showed that RRT initiation time was not associated with the length of ICU stay (MD − 0.06; 95% CI − 1.59 to 1.48; *P* = 0.94; *I*^2^ = 0%; 1008 participants; see Additional file 5d) or hospital stay (MD − 2.88; 95% CI − 6.57 to 0.81; *P* = 0.13; *I*^2^ = 41%; 1835 participants; see Additional file 5e).

### Mechanical ventilation-free days, RRT-free days and vasopressor-free days up to day 28

Eight studies reported mechanical ventilation-free days up to day 28 [14, 20, 22–26, 29]. Six studies reported RRT-free days up to day 28 [20, 22, 23, 25–27]. Four studies reported vasopressor-free days up to day 28 [14, 20, 22, 23]. Meta-analysis showed that there were no significant differences in terms of mechanical ventilation-free days at 28 days (MD 0.47; 95% CI − 0.42 to 1.36; *P* = 0.30; *I*^2^ = 31%; 4718 participants; see Additional file 5f), RRT-free days at 28 days (MD − 1.13; 95% CI − 2.36 to 0.10; *P* = 0.07; *I*^2^ = 21%; 1589 participants; see Additional file 5g), or vasopressor-free days at 28 days (MD 0.39; 95% CI − 0.48 to 1.25; *P* = 0.38; *I*^2^ = 0%; 4258 participants; see Additional file 5h) between the early-strategy group and the delayed-strategy group.

### Adverse events during treatment

**Fig. 4:**
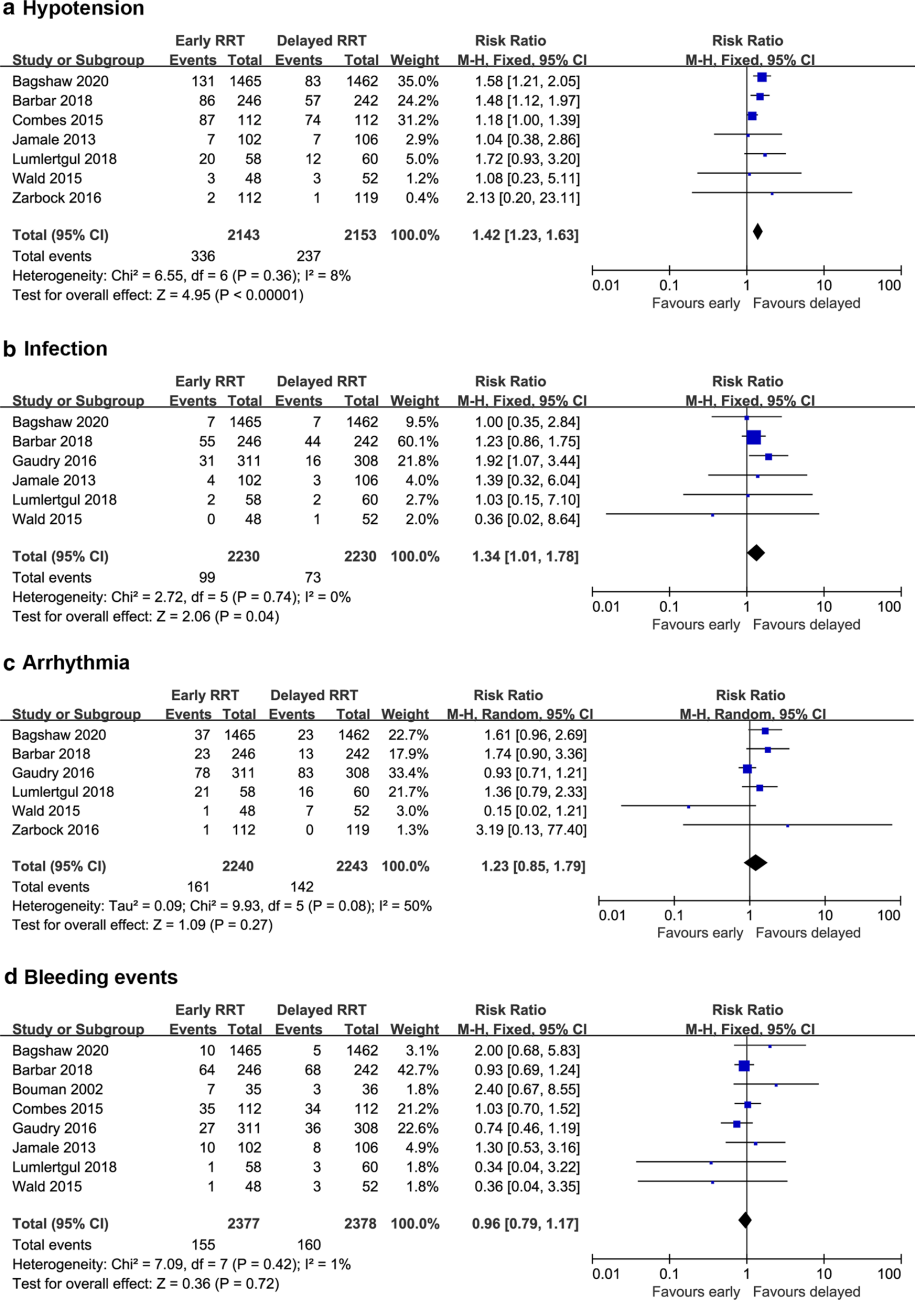
Forest plot of comparison: early RRT initiation group versus delayed RRT initiation group. **a** Hypotension; **b** infection; **c** arrhythmia; **d** bleeding events

Seven studies reported hypotension events [14, 22–25, 27, 28]. There were 336 patients (15.7%) who developed hypotension among 2143 patients in the early-strategy group and 237 patients (11.0%) who developed hypotension among 2153 patients in the delayed-strategy group. According to the results, early RRT initiation leads to more hypotension events than delayed RRT initiation (RR 1.42; 95% CI 1.23 to 1.63; *P* < 0.00001; *I*^2^ = 8%; Fig. 4a). Six studies involving 4460 patients reported the RRT-associated infection during treatments [14, 20, 22, 25, 27, 28]. Infection occurred 99 patients (4.4%) in the early-strategy group and 73 patients (3.3%) in the delayed-strategy group. Early RRT initiation resulted in a significantly higher incidence of RRT-associated infection events (RR 1.34; 95% CI 1.01 to 1.78; *P* = 0.04; *I*^2^ = 0%; Fig. 4b). In terms of arrhythmia and bleeding events, the pooled RRs were 1.23 (95% CI 0.85 to 1.79; *P* = 0.27; *I*^2^ = 50%; 4483 participants; 6 studies; Fig. 4c) and 0.96 (RR 0.96; 95% CI 0.79 to 1.17; *P* = 0.72; *I*^2^ = 1%; 4755 participants; 8 studies; Fig. 4d), respectively. There were no statistical differences between the two groups.

### Subgroup analyses and sensitivity analyses

From the subgroup analyses of the primary outcome, we found that the mean age of patients in each study (> 65 years or ≤ 65 years), the SOFA scores at administration (> 12 or ≤ 12), and the different criteria for early RRT initiation had no significant effect on 28-day all-cause mortality. Sensitivity analyses according to publish language (excluding the study published in Chinese), risk of bias (only including studies classified as low risk of bias), and publish year (removing studies published before 2010) did not alter the conclusion of the main analyses. The detailed results about subgroup analyses and sensitivity analyses are presented in Table 2 and Additional file 6.

**Table 2:** Results of sensitivity analyses and subgroup analyses

Group		No. of trials	No. of patients	Risk ratio (95% CI)	*P* value	Heterogeneity	
						*I*^2^, %	*P* value for *I*^2^
*Subgroup analyses*							
Mean age							
> 65 years		7 [20, 22, 24–26, 29, 30]	1627	0.99 (0.89, 1.11)	0.88	0	0.67
≤ 65 years		3 [14, 23, 27]	3251	1.02 (0.93, 1.12)	0.63	0	0.95
SOFA scores at administration							
> 12		3 [22, 24, 27]	819	0.95 (0.75, 1.20)	0.65	31	0.24
≤ 12		7 [14, 20, 23, 25, 26, 29, 30]	4059	1.02 (0.94, 1.10)	0.62	0	0.98
Criteria for early RRT initiation							
Approximately equal to stage 2 of the KDIGO classification		4 [14, 24, 27, 29]	3329	1.01 (0.92, 1.10)	0.89	4	0.37
Approximately equal to stage 3 of the KDIGO classification		2 [20, 22]	1107	1.00 (0.88, 1.15)	0.95	0	0.41
Other classification criteria		4 [23, 25, 26, 30]	442	1.05 (0.86, 1.29)	0.61	0	0.97
*Sensitivity analyses*							
Publish language (excluding the study published in Chinese)		9 [14, 20, 22–27, 29]	4818	1.01 (0.94, 1.08)	0.81	0	0.85
Risk of bias (only including low risk of bias studies)		8 [14, 20, 22–27]	4747	1.01 (0.94, 1.08)	0.86	0	0.81
Publish year (removing studies published before 2010)		9 [14, 20, 22–27, 30]	4807	1.01 (0.94, 1.08)	0.82	0	0.86

*CI* confidence interval, *SOFA* Sequential Organ Failure Assessment

## Discussion

This systematic review and meta-analysis included 11 studies comparing delayed versus early initiation of RRT for AKI in critically ill patients. The pooled results showed that early initiation of RRT was not associated with survival benefit in critically ill patients with AKI. The TSA results indicated a RRR of 15% or greater could be rejected with respect to 28-day, 90-day, or hospital mortality. In addition, early initiation of RRT could lead to unnecessary RRT exposure in some patients, resulting in a higher incidence of RRT-associated adverse events, including hypotension and infection.

Over the past few decades, RRT has become more sophisticated, with more modalities available, each with its own merits in particular situations [8]. RRT can be life-saving by correcting metabolic disorders in patients with severe acidosis and hyperkalemia, controlling disturbances of fluid metabolism in patients with severe pulmonary edema, and removing toxins and circulating inflammatory cytokines in patients with severe sepsis. We can learn from the inclusion criteria for each of the included studies that two studies included patients with sepsis, one study included patients with shock after cardiac surgery, and the other eight studies included mixed populations (see Additional file 7). Causes and pathophysiological mechanisms of AKI were highly variable in different studies, such as renal hypoperfusion, nephrotoxin exposure, ischemic reperfusion injury, and an increase in the level of circulating inflammatory cytokines. To our knowledge, the prognosis of RRT for AKI induced by different causes may be different. Moreover, the criteria for the initiation of RRT, the definition of AKI and RRT modalities existed great variations among the included studies. Therefore, we should be cautious with the results of this study.

So far, this is the only meta-analysis including the STARRT‑AKI trial [14]. Our results were consistent with the results of most previous meta-analyses except three, which reported early initiation of RRT may have significant benefit on survival [9, 31, 32]. However, a considerable proportion of the included studies in these three reviews were non-RCTs, meaning that the data were prone to confounding factors. Another two meta-analyses with TSA of RCTs conducted by Moreira et al. and Feng et al. failed to establish sufficient and conclusive evidences, because the cumulative *Z *curve did not cross the conventional boundary, the trial sequential monitoring boundary and the futility boundary, and the required information size was not reached [33, 34]. However, in our meta-analysis, the cumulative *Z* curve crossed the futility boundaries, suggesting the results that early initiation of RRT was not associated with a lower mortality were reliable.

Hemodynamic instability is a common complication during RRT, which can increase hospital mortality and limit kidney recovery [35, 36]. Many factors contribute to hemodynamic instability, including excessive ultrafiltration, rapid osmotic/oncotic shifts, decreased cardiac output, and decreased peripheral resistance [37]. The incidence of hypotension was 15.7% and 11.0% in the early-strategy group and in the delayed-strategy group, respectively. We can learn from Table 1 that studies which reported hypotension events all involved intermittent hemodialysis (IHD), which was more likely to result in hemodynamic instability than CRRT. A significant difference detected between the two groups may be due to more patients in the early-strategy group exposure to RRT (2468 of 2539 patients) compared with the delayed-strategy group (1591 of 2547 patients). However, none of the included studies reported the mode of RRT when the hypotension occurred. Therefore, we failed to find the association between RRT mode and hypotension in this meta-analysis. There were no statistical significances in hospital mortality and kidney recovery between the two groups, but hospital mortality and 90-day RRT dependence rates were higher in the early RRT group than the delayed RRT group. A remarkable higher incidence of RRT-associated infection events was also found in the early RRT group. Patients treated with RRT are more susceptible to infection, as they are exposure to catheters and invasive treatments [38, 39]. Moreover, RRT may enhance the elimination of antibiotics, leading to suboptimal antibiotic concentrations [40].

In this meta-analysis, only 62.5% patients in the delayed-strategy group received RRT. Although fewer patients received RRT in the delayed-strategy group compared to the early-strategy group, the clinical outcomes were comparable between the two groups. In addition, our results showed that delayed RRT initiation could reduce the incidence of RRT-associated adverse events. Undoubtedly, unnecessary RRT will increase the workload of medical staff, augment treatment costs, and waste health resources. Therefore, it is reasonable to assume that delayed initiation of RRT is a preferable approach for critically ill patients with AKI.

As shown in Table 1, the criteria for initiating RRT and definition of AKI were associated with great variations among the included studies. Timing of RRT initiation was determined by AKI stage, biochemical marker level or urine output. AKI was defined by the RIFLE (risk, injury, failure, loss, and end-stage) criteria, the AKIN (AKI Network) criteria, or the KDIGO (Kidney Disease: Improving Global Outcomes) criteria. One problem was that patients who were classified into the early-strategy group in one study might be classified into the delayed-strategy group in another. Despite the definition of early RRT had differences among the included studies, the criteria for early RRT initiation were similar in some studies. And the subgroup analyses based on the criteria for early RRT initiation also showed that early RRT could not decrease 28-day all-cause mortality compared with delayed RRT. Although there were differences in the definition of the delayed RRT, most of studies initiated RRT when patients were with severe complications such as severe pulmonary edema, severe acidosis, and severe hyperkalemia. It is reasonable for us to assume that the optimal timing of initiating RRT is when patients are with severe complications. It is also worth noting that although there are a variety of criteria for initiating RRT, it is mainly based on renal function indicators at present. RRT cannot only influence renal function, but also have an effect on other organs, such as liver function, cardiac function, and so on [41, 42]. Perhaps establishing a scoring system based on systemic multi-organ functions to find the best cutoff time initiating RRT is the way forward, just like Sequential Organ Failure Assessment (SOFA) score and Acute Physiology and Chronic Health Evaluation II (APACHE II) score.

The strengths of our study are as follows: First, we only included RCTs and most of the included studies were assessed as low risk of bias. Second, we comprehensively evaluated the effect of RRT initiation timing on clinical outcomes, including mortality, renal function recovery, various adverse events, and so on. Third, we performed TSA to determine whether the evidences in our research were reliable. Notwithstanding the aforementioned, there are several limitations in our study. The main limitation is the criteria for the initiation of RRT had great variations among the included studies. Second, we did not perform subgroup analyses according to RRT modalities, delivered dialysis dose. The choice of the RRT modality in most included studies were prescribed and monitored according to national guidelines. Some patients received CRRT at the outset, but may switch to other RRT modalities depending to their conditions. We tried to find whether the choice of RRT modality may influence the results. However, since this was a secondary analysis study, the individual patient data was not available. We cannot further analysis the effect of RRT modality on outcomes.

## Conclusions

This meta-analysis suggested that early initiation of RRT was not associated with survival benefit in critically ill patients with AKI. In addition, early initiation of RRT could lead to unnecessary RRT exposure in some patients, resulting in a waste of health resources and a higher incidence of RRT-associated adverse events. Maybe, only critically ill patients with a clear and hard indication, such as severe acidosis, pulmonary edema, and hyperkalemia could benefit from early initiation of RRT.

## Key messages

1. Early initiation of RRT was not associated with survival benefit in critically ill patients with AKI.
2. Early initiation of RRT could lead to unnecessary RRT exposure in some patients, resulting in a waste of health resources and a higher incidence of RRT-associated adverse events, including hypotension and infection.
3. Delayed initiation of RRT might be safe in the absence of life-threatening conditions, such as acute pulmonary edema, severe acidosis, and severe hyperkalemia.

## Supplementary Information

**Additional file 1:** PRISMA checklist.

**Additional file 2:** Search strategy terms and results.

**Additional file 3:** Risk of bias graph and risk of bias summary graph.

**Additional file 4:** Funnel plots: a. Funnel plot to evaluate potential publication bias for 28-day mortality; b. Funnel plot to evaluate potential publication bias for 90-day mortality; c. Funnel plot to evaluate potential publication bias for ICU mortality; d. Funnel plot to evaluate potential publication bias for hospital mortality.

**Additional file 5:** Forest plot of the secondary outcomes: a. Number of patients who received RRT; b. RRT dependence among survivors at 28 days; c. RRT dependence among survivors at 90 days; d. The length of ICU stay; e. The length of hospital stay; f. Mechanical ventilation-free days at 28 days; g. RRT-free days at 28 days; h. Vasopressor-free days at 28 days.

**Additional file 6:** Forest plot of the subgroup analyses and sensitivity analyses: a.Subgroup analyses for 28-day mortality divided by the mean age of participants in each study; b. Subgroup analyses for 28-day mortality divided by the SOFA scores at administration; c. Subgroup analyses for 28-day mortality divided by the criteria for early RRT initiation; d. Sensitivity analyses according to publish language (excluding the study published in Chinese); e. Sensitivity analyses according to risk of bias (only including studies classified as low risk of bias); f. Sensitivity analyses according to publish year (removing studies published before 2010).

**Additional file 7:** The inclusion criteria for each of the included studies in this meta-analysis.

## Data Availability

All data generated or analyzed during this study are included in this published article (and its supplementary information files).

## Abbreviations

AKI: Acute kidney injury
APACHE II: Acute Physiology and Chronic Health Evaluation II
AKIN: Acute kidney injury network
ICU: Intensive care unit
IHD: Intermittent hemodialysis
KDIGO: Kidney disease, improving global outcomes
PRISMA: Preferred reporting items for systematic reviews and meta-analyses
RRR: Relative risk reduction
RRT: Renal replacement therapy
RCTs: Randomized clinical trials
RIFLE: Risk, injury, failure, loss, and end-stage
SOFA: Sequential Organ Failure Assessment
TSA: Trial sequential analysis
