# Supplementary material for: Timing of renal replacement therapy initiation for acute kidney injury in critically ill patients: a systematic review of randomized clinical trials with meta-analysis and trial sequential analysis
Source: Crit Care. 2021 Jan 6;25:15. doi: 10.1186/s13054-020-03451-y (PMC7789484; doi:10.1186/s13054-020-03451-y)
Supplement: Supplementary file 2 — Additional file 2: Search strategy terms and results. [file 13054_2020_3451_MOESM2_ESM.docx]

**Search strategy terms and results**

| PUBMED, searched 20 July 2020 | | |
| --- | --- | --- |
| 1 | Acute kidney injury [MeSH majr] OR acute kidney injury[tiab] OR acute renal failure[tiab] | 57,732 |
| 2 | Renal replacement therapy [MeSH majr] OR renal replacement therapy [tiab] OR  renal-replacement therapy [tiab] OR dialysis [tiab] OR hemodialysis [tiab] OR hemofiltration [tiab] | 241,068 |
| 3 | Time to treatment [MeSH] OR Time factors [MeSH] OR early [tiab] OR earlier [tiab] OR time [tiab] OR timing [tiab] OR accelerated [tiab] OR accelerating [tiab] OR acceleration [tiab] OR late [tiab] OR delayed [tiab] | 5,802,343 |
| 4 | Intensive Care Unit [All Fields] OR critically ill [All Fields] OR critical illness [All Fields] OR intensive care [All Fields] OR ICU [All Fields] OR critical care [All Fields] | 512,304 |
| 5 | Randomized controlled trial[pt] OR controlled clinical trial[pt] OR randomized[tiab] OR placebo[tiab] OR drug therapy[sh] OR randomly[tiab] OR **trial**[tiab] **OR groups**[tiab] | 4,811,670 |
| 6 | Animals [MeSH] NOT humans [MeSH] | 4,719,527 |
| 7 | #5 NOT #6 | 4,178,874 |
| 4 | #1 AND # 2 AND # 3 AND # 4 AND # 7 | 651 |
