# Supplementary figures and images for: Timing of renal replacement therapy initiation for acute kidney injury in critically ill patients: a systematic review of randomized clinical trials with meta-analysis and trial sequential analysis
Source: Crit Care. 2021 Jan 6;25:15. doi: 10.1186/s13054-020-03451-y (PMC7789484; doi:10.1186/s13054-020-03451-y)

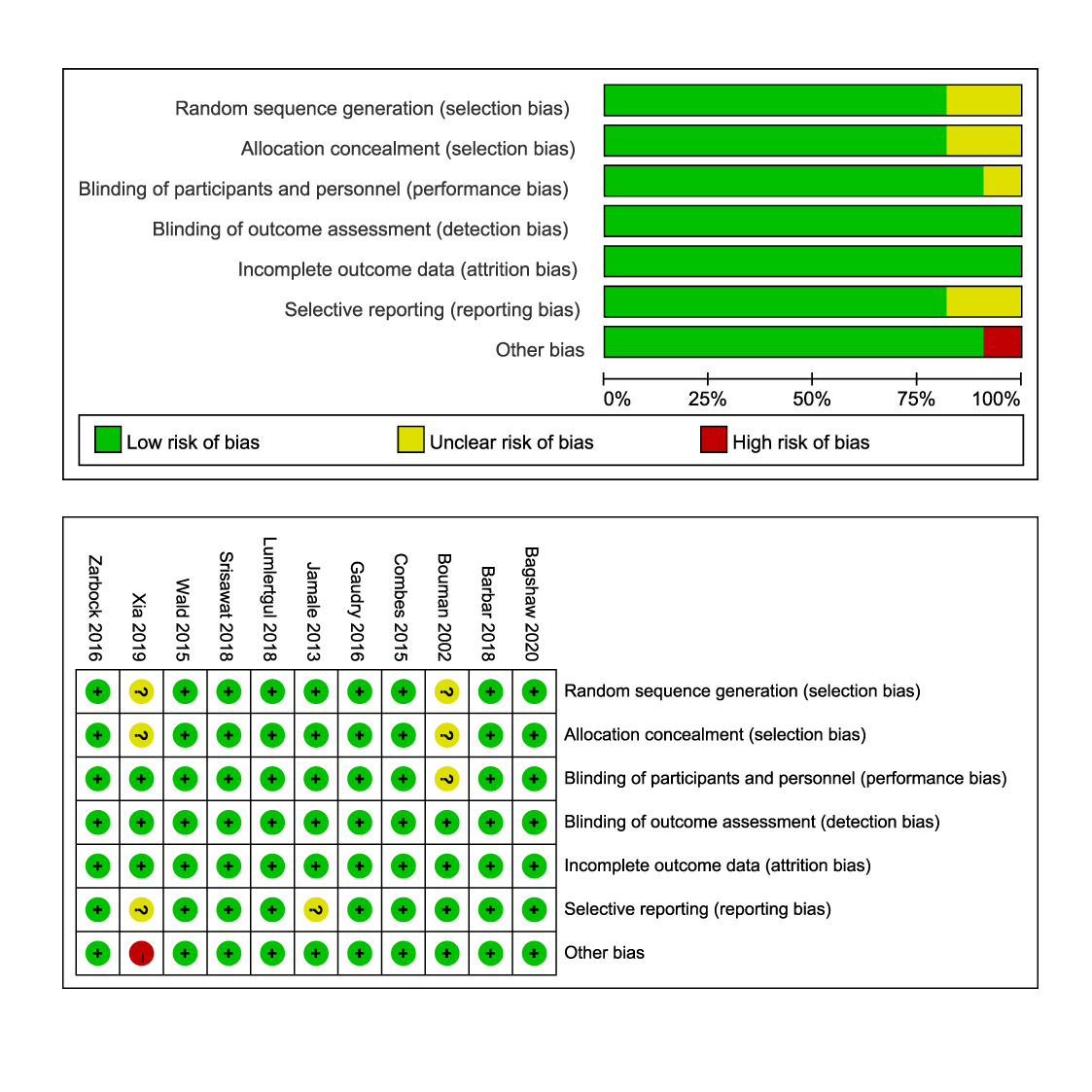

Supplement: Supplementary file 3 — Additional file 3: Risk of bias graph and risk of bias summary graph. [file 13054_2020_3451_MOESM3_ESM.tif]
