# Supplementary material for: Timing of renal replacement therapy initiation for acute kidney injury in critically ill patients: a systematic review of randomized clinical trials with meta-analysis and trial sequential analysis
Source: Crit Care. 2021 Jan 6;25:15. doi: 10.1186/s13054-020-03451-y (PMC7789484; doi:10.1186/s13054-020-03451-y)

a. **Funnel plot to evaluate potential publication bias for 28-day mortality**

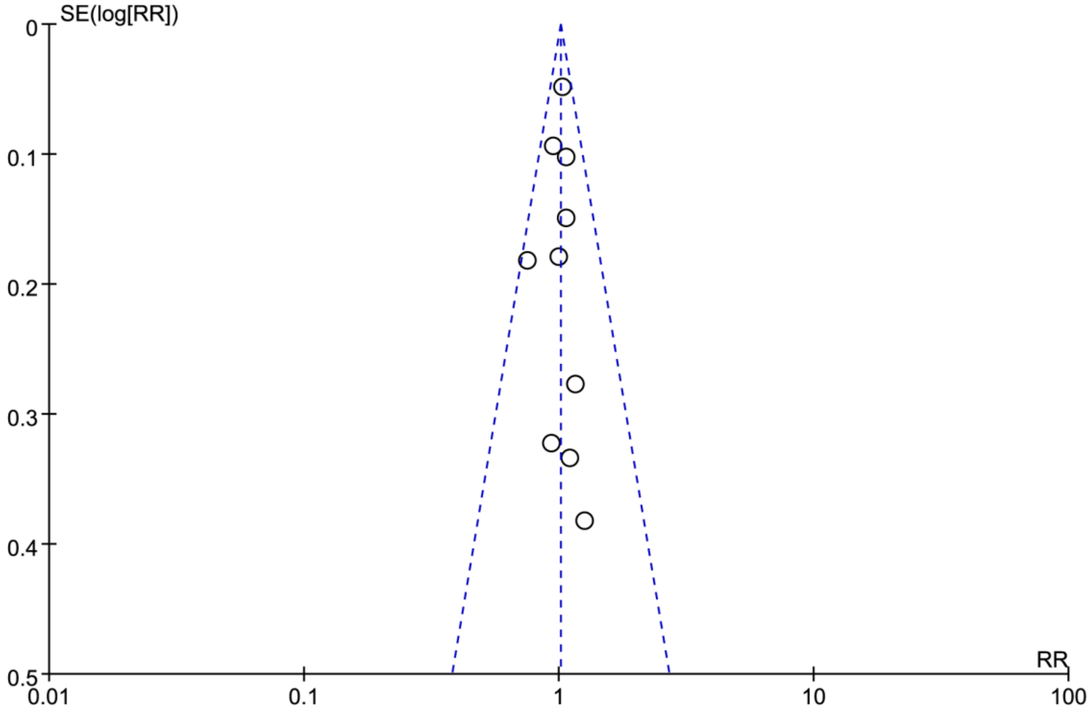

b. **Funnel plot to evaluate potential publication bias for 90-day mortality**

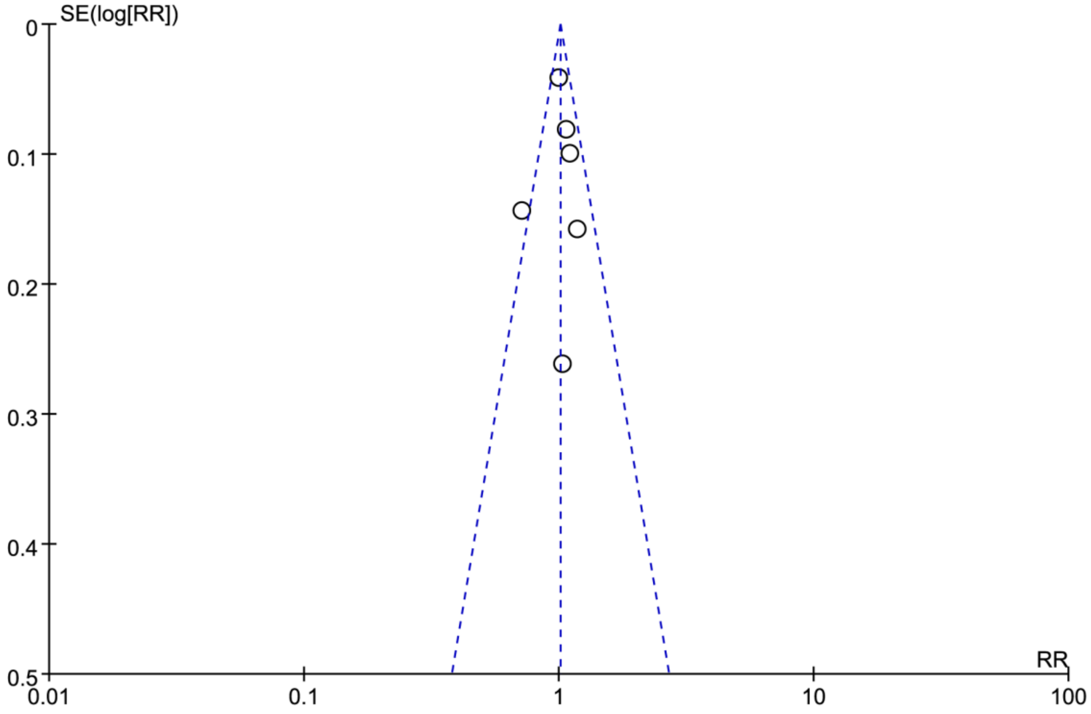

c. Funnel plot to evaluate potential publication bias for ICU mortality

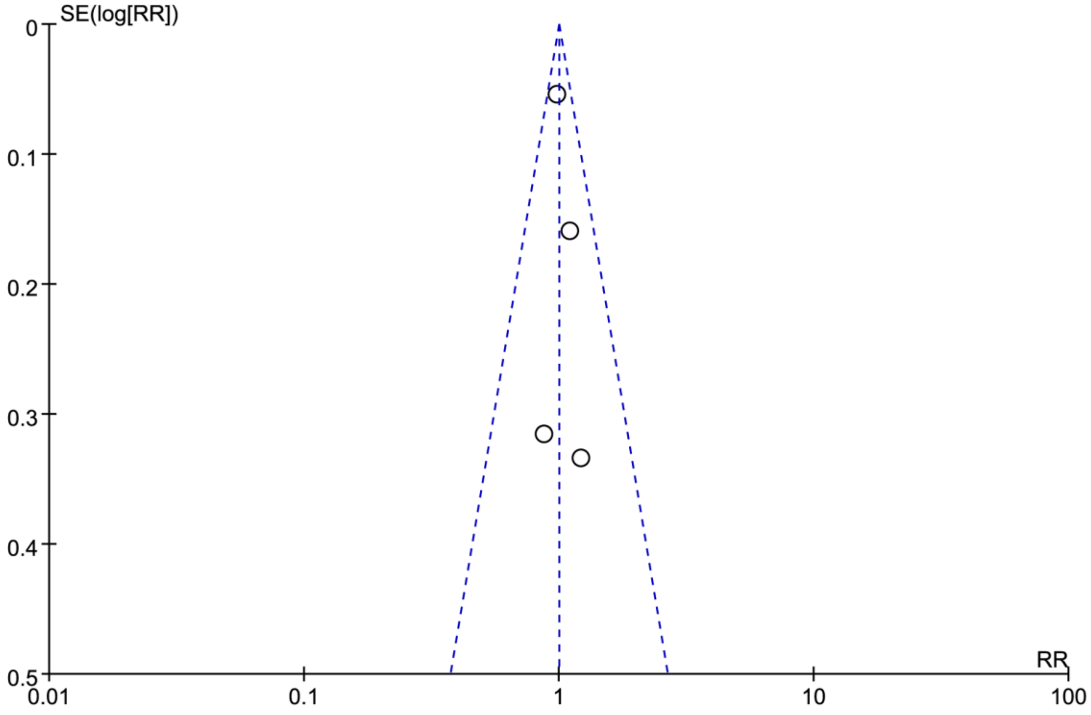

d. Funnel plot to evaluate potential publication bias for hospital mortality

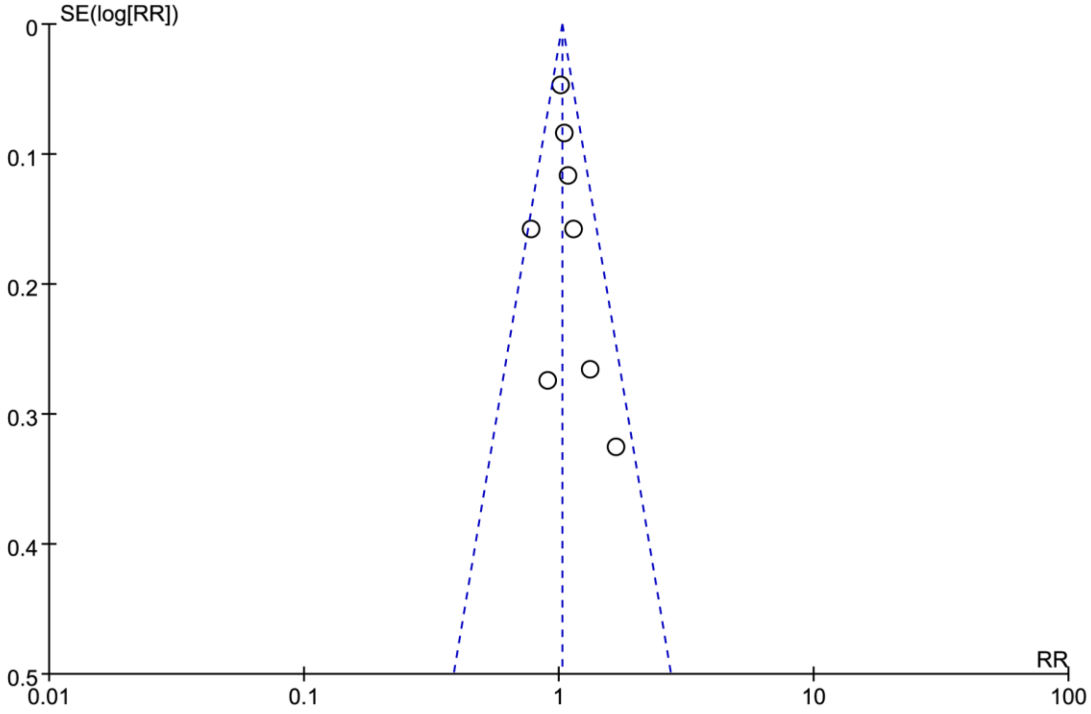

Supplement: Supplementary file 4 — Additional file 4: Funnel plots: a. Funnel plot to evaluate potential publication bias for 28-day mortality; b. Funnel plot to evaluate potential publication bias for 90-day mortality; c. Funnel plot to evaluate potential publication bias for ICU mortality; d. Funnel plot to evaluate potential publication bias for hospital mortality. [file 13054_2020_3451_MOESM4_ESM.pdf]
