# Supplementary material for: Timing of renal replacement therapy initiation for acute kidney injury in critically ill patients: a systematic review of randomized clinical trials with meta-analysis and trial sequential analysis
Source: Crit Care. 2021 Jan 6;25:15. doi: 10.1186/s13054-020-03451-y (PMC7789484; doi:10.1186/s13054-020-03451-y)

a. Number of patients who received RRT

Study or Subgroup	Early RRT Events	Early RRT Total	Delayed RRT Events	Delayed RRT Total	Weight	Risk Ratio M-H, Random, 95% CI
Bagshaw 2020	1418	1465	903	1462	10.9%	1.57 [1.50, 1.63]
Barbar 2018	239	246	149	242	10.4%	1.58 [1.43, 1.75]
Bouman 2002	35	35	30	36	9.9%	1.20 [1.03, 1.40]
Combes 2015	111	112	64	112	9.8%	1.73 [1.48, 2.04]
Gaudry 2016	305	311	157	308	10.4%	1.92 [1.72, 2.15]
Jamale 2013	93	102	88	106	10.4%	1.10 [0.99, 1.22]
Lumlertgul 2018	57	58	45	60	9.9%	1.31 [1.13, 1.52]
Srisawat 2018	20	20	8	20	4.9%	2.41 [1.43, 4.07]
Wald 2015	48	48	33	52	9.2%	1.57 [1.27, 1.93]
Xia 2019	30	30	6	30	3.5%	4.69 [2.37, 9.31]
Zarbock 2016	112	112	108	119	10.8%	1.10 [1.04, 1.17]
**Total (95% CI)**		**2539**		**2547**	**100.0%**	**1.52 [1.30, 1.78]**
Total events	2468		1591			

Heterogeneity: Tau² = 0.06; Chi² = 217.43, df = 10 (P < 0.00001); I² = 95%
Test for overall effect: Z = 5.31 (P < 0.00001)

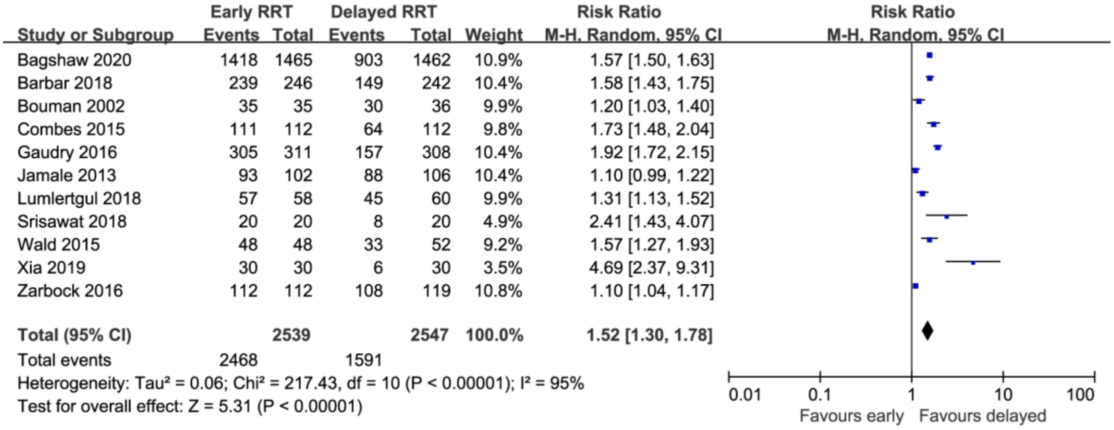

b. RRT dependence among survivors at 28 days

Study or Subgroup	Early RRT Events	Early RRT Total	Delayed RRT Events	Delayed RRT Total	Weight	Risk Ratio M-H, Random, 95% CI
Barbar 2018	17	134	17	140	26.0%	1.04 [0.56, 1.96]
Bouman 2002	1	17	0	22	2.5%	3.83 [0.17, 88.62]
Combes 2015	6	72	2	72	8.4%	3.00 [0.63, 14.37]
Gaudry 2016	22	179	17	178	27.0%	1.29 [0.71, 2.34]
Srisawat 2018	1	10	6	11	5.9%	0.18 [0.03, 1.27]
Zarbock 2016	18	78	26	71	30.2%	0.63 [0.38, 1.05]
**Total (95% CI)**		**490**		**494**	**100.0%**	**0.97 [0.58, 1.60]**
Total events	65		68			

Heterogeneity: Tau² = 0.15; Chi² = 9.18, df = 5 (P = 0.10); I² = 46%
Test for overall effect: Z = 0.13 (P = 0.89)

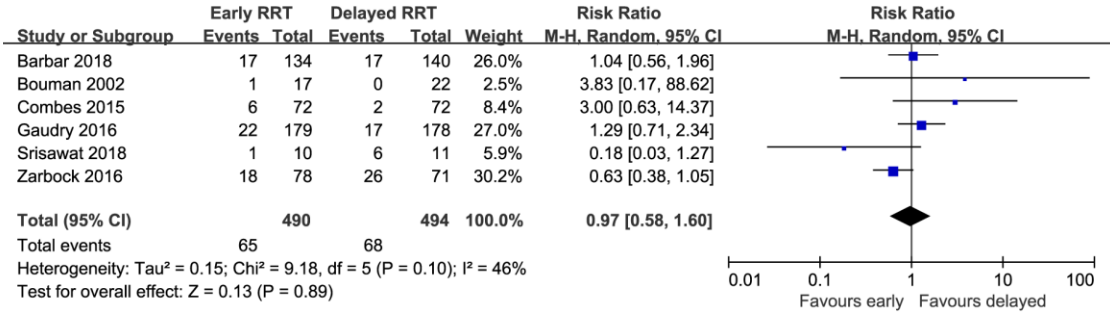

c. RRT dependence among survivors at 90 days

Study or Subgroup	Early RRT Events	Early RRT Total	Delayed RRT Events	Delayed RRT Total	Weight	Risk Ratio M-H, Random, 95% CI
Bagshaw 2020	85	814	49	815	60.1%	1.74 [1.24, 2.43]
Barbar 2018	2	101	3	110	9.2%	0.73 [0.12, 4.26]
Combes 2015	0	61	0	69		Not estimable
Wald 2015	0	30	2	33	3.5%	0.22 [0.01, 4.39]
Zarbock 2016	9	67	8	53	27.2%	0.89 [0.37, 2.15]
**Total (95% CI)**		**1073**		**1080**	**100.0%**	**1.24 [0.70, 2.21]**
Total events	96		62			

Heterogeneity: Tau² = 0.11; Chi² = 4.29, df = 3 (P = 0.23); I² = 30%
Test for overall effect: Z = 0.75 (P = 0.46)

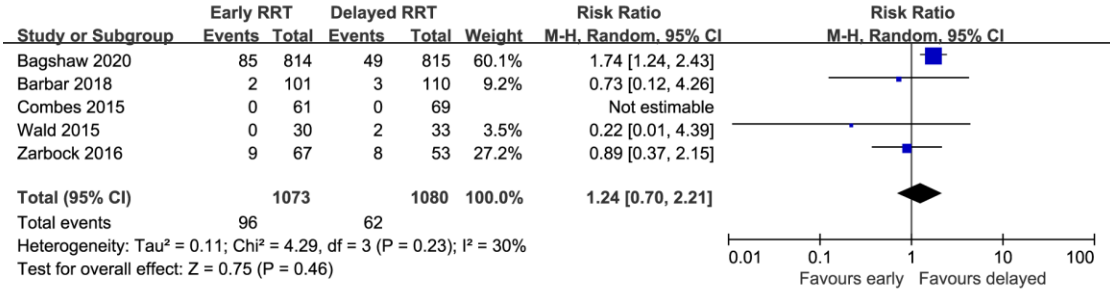

d. The length of ICU stay

Study or Subgroup	Early RRT Mean	Early RRT SD	Early RRT Total	Delayed RRT Mean	Delayed RRT SD	Delayed RRT Total	Weight	Mean Difference IV, Fixed, 95% CI
Barbar 2018	11	11.1	246	10	10.4	242	64.8%	1.00 [-0.91, 2.91]
Bouman 2002	13	11.9	35	13.5	11.7	36	7.8%	-0.50 [-5.99, 4.99]
Lumlertgul 2018	12	14.1	58	13.5	14.8	60	8.7%	-1.50 [-6.71, 3.71]
Wald 2015	11	15.9	48	13.5	17.8	52	5.4%	-2.50 [-9.11, 4.11]
Zarbock 2016	19	14.8	112	22	17.8	119	13.3%	-3.00 [-7.21, 1.21]
**Total (95% CI)**			**499**			**509**	**100.0%**	**-0.06 [-1.59, 1.48]**

Heterogeneity: Chi² = 3.90, df = 4 (P = 0.42); I² = 0%
Test for overall effect: Z = 0.07 (P = 0.94)

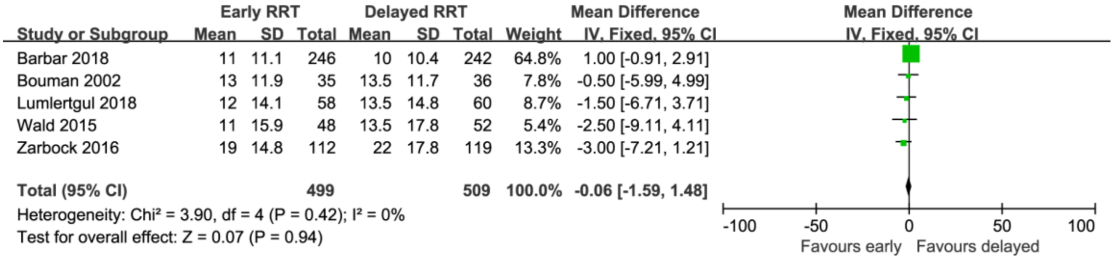

e. The length of hospital stay

Study or Subgroup	Early RRT Mean	Early RRT SD	Early RRT Total	Delayed RRT Mean	Delayed RRT SD	Delayed RRT Total	Weight	Mean Difference IV, Random, 95% CI
Barbar 2018	29.3	29.1	246	34.7	41.3	242	18.6%	-5.40 [-11.75, 0.95]
Bouman 2002	27	20.7	35	35.5	38.5	36	5.7%	-8.50 [-22.82, 5.82]
Gaudry 2016	31	46.9	311	28.4	35.5	308	18.0%	2.60 [-3.95, 9.15]
Jamale 2013	5.6	3.4	102	7.5	5.8	106	39.4%	-1.90 [-3.19, -0.61]
Lumlertgul 2018	29.7	35.5	58	32.6	34.9	60	7.0%	-2.90 [-15.61, 9.81]
Wald 2015	32.1	41.4	48	28.9	28.1	52	6.0%	3.20 [-10.78, 17.18]
Zarbock 2016	44.2	41.9	112	64.6	70.6	119	5.4%	-20.40 [-35.27, -5.53]
**Total (95% CI)**			**912**			**923**	**100.0%**	**-2.88 [-6.57, 0.81]**

Heterogeneity: Tau² = 8.58; Chi² = 10.24, df = 6 (P = 0.11); I² = 41%

Test for overall effect: Z = 1.53 (P = 0.13)

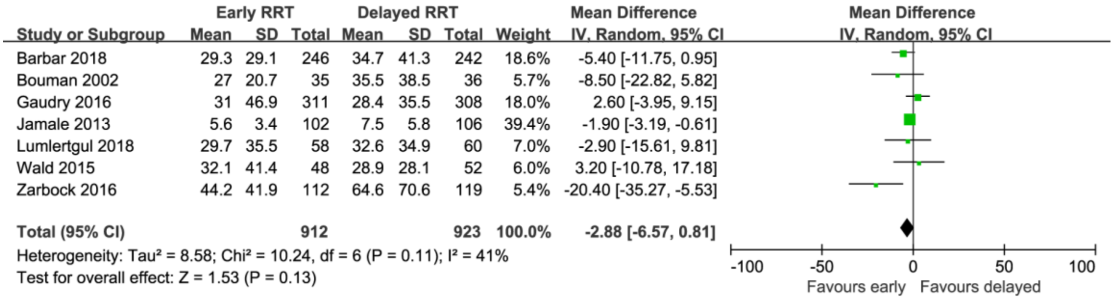

f. Mechanical ventilation-free days at 28 days

Study or Subgroup	Early RRT Mean	Early RRT SD	Early RRT Total	Delayed RRT Mean	Delayed RRT SD	Delayed RRT Total	Weight	Mean Difference IV, Random, 95% CI
Bagshaw 2020	13	17.8	1465	12	17.8	1462	23.1%	1.00 [-0.29, 2.29]
Barbar 2018	8.5	9.9	246	9.6	10.4	242	15.8%	-1.10 [-2.90, 0.70]
Bouman 2002	15	11.9	35	9.6	10.4	36	2.8%	5.40 [0.20, 10.60]
Combes 2015	1	3.7	112	1	4.4	112	27.4%	0.00 [-1.06, 1.06]
Gaudry 2016	9.3	11	311	8.9	10.8	308	16.8%	0.40 [-1.32, 2.12]
Lumlertgul 2018	8.6	11.9	58	7	10.8	60	4.3%	1.60 [-2.50, 5.70]
Srisawat 2018	10.7	12.4	20	5	9.6	20	1.6%	5.70 [-1.17, 12.57]
Zarbock 2016	9.3	11	112	8.9	10.8	119	8.2%	0.40 [-2.41, 3.21]
**Total (95% CI)**			**2359**			**2359**	**100.0%**	**0.47 [-0.42, 1.36]**

Heterogeneity: Tau² = 0.46; Chi² = 10.18, df = 7 (P = 0.18); I² = 31%

Test for overall effect: Z = 1.03 (P = 0.30)

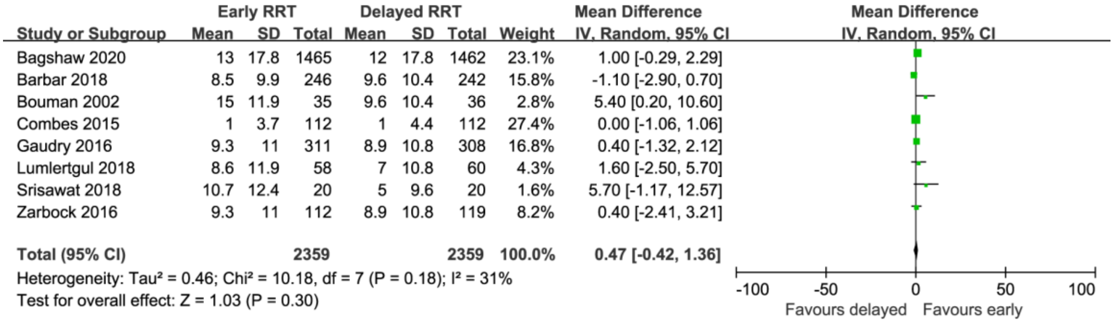

g. RRT-free days at 28 days

Study or Subgroup	Early RRT Mean	Early RRT SD	Early RRT Total	Delayed RRT Mean	Delayed RRT SD	Delayed RRT Total	Weight	Mean Difference IV, Fixed, 95% CI
Barbar 2018	13.4	11.4	246	14.8	11.8	242	35.5%	-1.40 [-3.46, 0.66]
Combes 2015	17	17.8	112	23	20.7	112	5.9%	-6.00 [-11.06, -0.94]
Gaudry 2016	12.6	12.1	311	13.6	13	308	38.4%	-1.00 [-2.98, 0.98]
Lumlertgul 2018	6.9	10.1	58	7.8	12.1	60	9.3%	-0.90 [-4.92, 3.12]
Srisawat 2018	9.4	11.3	20	10	13.9	20	2.4%	-0.60 [-8.45, 7.25]
Wald 2015	10.9	11.6	48	8.5	9.8	52	8.4%	2.40 [-1.83, 6.63]
**Total (95% CI)**			**795**			**794**	**100.0%**	**-1.13 [-2.36, 0.10]**

Heterogeneity: Chi² = 6.36, df = 5 (P = 0.27); I² = 21%

Test for overall effect: Z = 1.81 (P = 0.07)

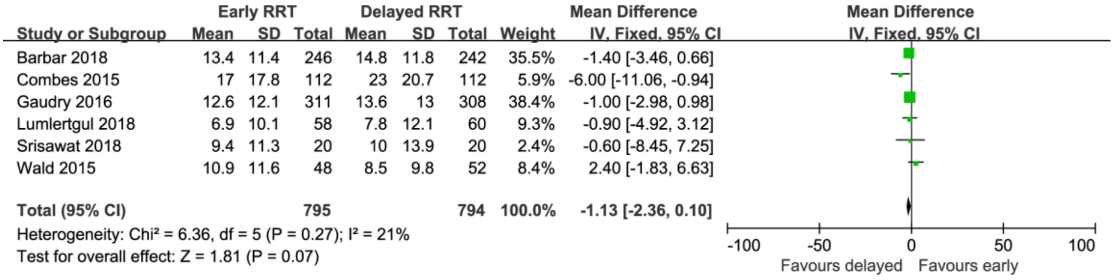

h. Vasopressor-free days at 28 days

Study or Subgroup	Early RRT Mean	Early RRT SD	Early RRT Total	Delayed RRT Mean	Delayed RRT SD	Delayed RRT Total	Weight	Mean Difference IV, Fixed, 95% CI
Bagshaw 2020	21	19.3	1465	20	19.3	1462	38.3%	1.00 [-0.40, 2.40]
Barbar 2018	13.6	11.6	246	13.8	11.5	242	17.8%	-0.20 [-2.25, 1.85]
Combes 2015	3	7.4	112	3	5.9	112	24.3%	0.00 [-1.75, 1.75]
Gaudry 2016	13.4	12.4	311	13.2	12.4	308	19.6%	0.20 [-1.75, 2.15]
**Total (95% CI)**			**2134**			**2124**	**100.0%**	**0.39 [-0.48, 1.25]**

Heterogeneity: Chi² = 1.28, df = 3 (P = 0.73); I² = 0%

Test for overall effect: Z = 0.87 (P = 0.38)

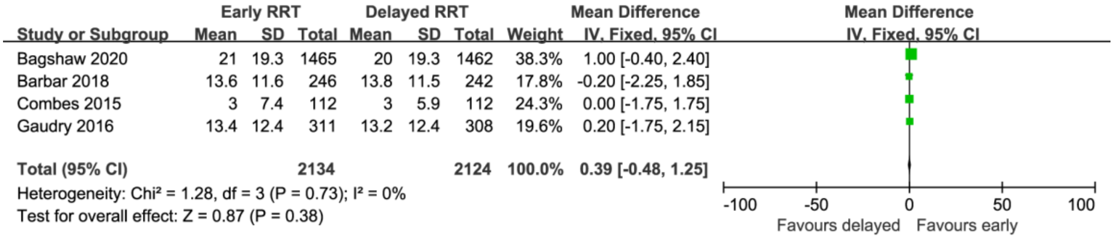

Supplement: Supplementary file 5 — Additional file 5: Forest plot of the secondary outcomes: a. Number of patients who received RRT; b. RRT dependence among survivors at 28 days; c. RRT dependence among survivors at 90 days; d. The length of ICU stay; e. The length of hospital stay; f. Mechanical ventilation-free days at 28 days; g. RRT-free days at 28 days; h. Vasopressor-free days at 28 days. [file 13054_2020_3451_MOESM5_ESM.pdf]
