# Supplementary material for: Timing of renal replacement therapy initiation for acute kidney injury in critically ill patients: a systematic review of randomized clinical trials with meta-analysis and trial sequential analysis
Source: Crit Care. 2021 Jan 6;25:15. doi: 10.1186/s13054-020-03451-y (PMC7789484; doi:10.1186/s13054-020-03451-y)

a. Subgroup analyses for 28-day mortality divided by the mean age of participants in each study

	Early RRT		Delayed RRT			Risk Ratio
Study or Subgroup	Events	Total	Events	Total	Weight	M-H, Fixed, 95% CI
**1.5.1 age>65 years group**						
Barbar 2018	111	246	102	242	11.1%	1.07 [0.87, 1.31]
Bouman 2002	11	35	9	36	1.0%	1.26 [0.59, 2.66]
Gaudry 2016	129	311	134	308	14.5%	0.95 [0.79, 1.15]
Lumlertgul 2018	36	58	35	60	3.7%	1.06 [0.79, 1.43]
Srisawat 2018	10	20	9	20	1.0%	1.11 [0.58, 2.14]
Xia 2019	15	30	13	30	1.4%	1.15 [0.67, 1.99]
Zarbock 2016	34	112	48	119	5.0%	0.75 [0.53, 1.07]
**Subtotal (95% CI)**		**812**		**815**	**37.7%**	**0.99 [0.89, 1.11]**
Total events	346		350			
Heterogeneity: Chi² = 4.06, df = 6 (P = 0.67); I² = 0%						
Test for overall effect: Z = 0.15 (P = 0.88)						
**1.5.2 age≤65 years group**						
Bagshaw 2020	538	1465	523	1462	56.5%	1.03 [0.93, 1.13]
Combes 2015	40	112	40	112	4.3%	1.00 [0.70, 1.42]
Wald 2015	13	48	15	52	1.6%	0.94 [0.50, 1.76]
**Subtotal (95% CI)**		**1625**		**1626**	**62.3%**	**1.02 [0.93, 1.12]**
Total events	591		578			
Heterogeneity: Chi² = 0.09, df = 2 (P = 0.95); I² = 0%						
Test for overall effect: Z = 0.48 (P = 0.63)						
**Total (95% CI)**		**2437**		**2441**	**100.0%**	**1.01 [0.94, 1.09]**
Total events	937		928			
Heterogeneity: Chi² = 4.25, df = 9 (P = 0.89); I² = 0%						
Test for overall effect: Z = 0.30 (P = 0.77)						
Test for subgroup differences: Chi² = 0.18, df = 1 (P = 0.67), I² = 0%						

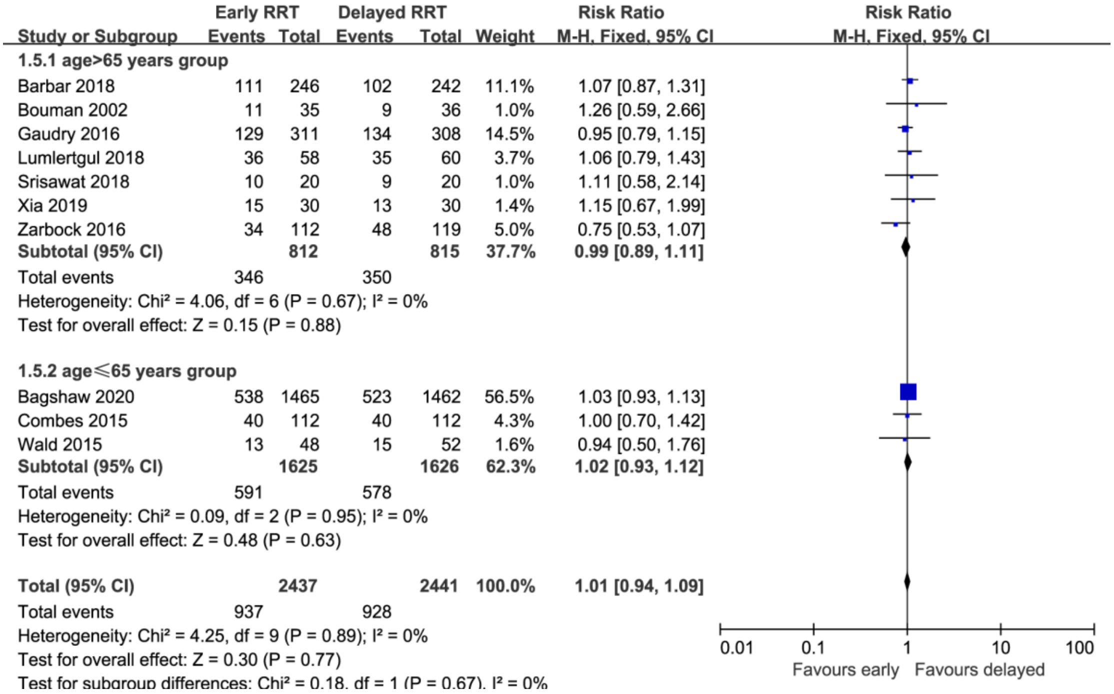

b. Subgroup analyses for 28-day mortality divided by the SOFA scores at administration

	Early RRT		Delayed RRT			Risk Ratio
Study or Subgroup	Events	Total	Events	Total	Weight	M-H, Random, 95% CI
**1.6.1 SOFA score at randomisation>12 group**						
Zarbock 2016	111	246	102	242	12.3%	1.07 [0.87, 1.31]
Wald 2015	13	48	15	52	1.3%	0.94 [0.50, 1.76]
Barbar 2018	34	112	48	119	4.0%	0.75 [0.53, 1.07]
**Subtotal (95% CI)**		**406**		**413**	**17.5%**	**0.95 [0.75, 1.20]**
Total events	158		165			
Heterogeneity: Tau² = 0.01; Chi² = 2.89, df = 2 (P = 0.24); I² = 31%						
Test for overall effect: Z = 0.46 (P = 0.65)						
**1.6.2 SOFA score at randomisation≤12 group**						
Xia 2019	15	30	13	30	1.7%	1.15 [0.67, 1.99]
Srisawat 2018	10	20	9	20	1.2%	1.11 [0.58, 2.14]
Lumlertgul 2018	36	58	35	60	5.8%	1.06 [0.79, 1.43]
Gaudry 2016	129	311	134	308	14.9%	0.95 [0.79, 1.15]
Combes 2015	40	112	40	112	4.0%	1.00 [0.70, 1.42]
Bouman 2002	11	35	9	36	0.9%	1.26 [0.59, 2.66]
Bagshaw 2020	538	1465	523	1462	54.1%	1.03 [0.93, 1.13]
**Subtotal (95% CI)**		**2031**		**2028**	**82.5%**	**1.02 [0.94, 1.10]**
Total events	779		763			
Heterogeneity: Tau² = 0.00; Chi² = 1.19, df = 6 (P = 0.98); I² = 0%						
Test for overall effect: Z = 0.50 (P = 0.62)						
**Total (95% CI)**		**2437**		**2441**	**100.0%**	**1.01 [0.94, 1.09]**
Total events	937		928			
Heterogeneity: Tau² = 0.00; Chi² = 4.25, df = 9 (P = 0.89); I² = 0%						
Test for overall effect: Z = 0.35 (P = 0.72)						
Test for subgroup differences: Chi² = 0.35, df = 1 (P = 0.55), I² = 0%						

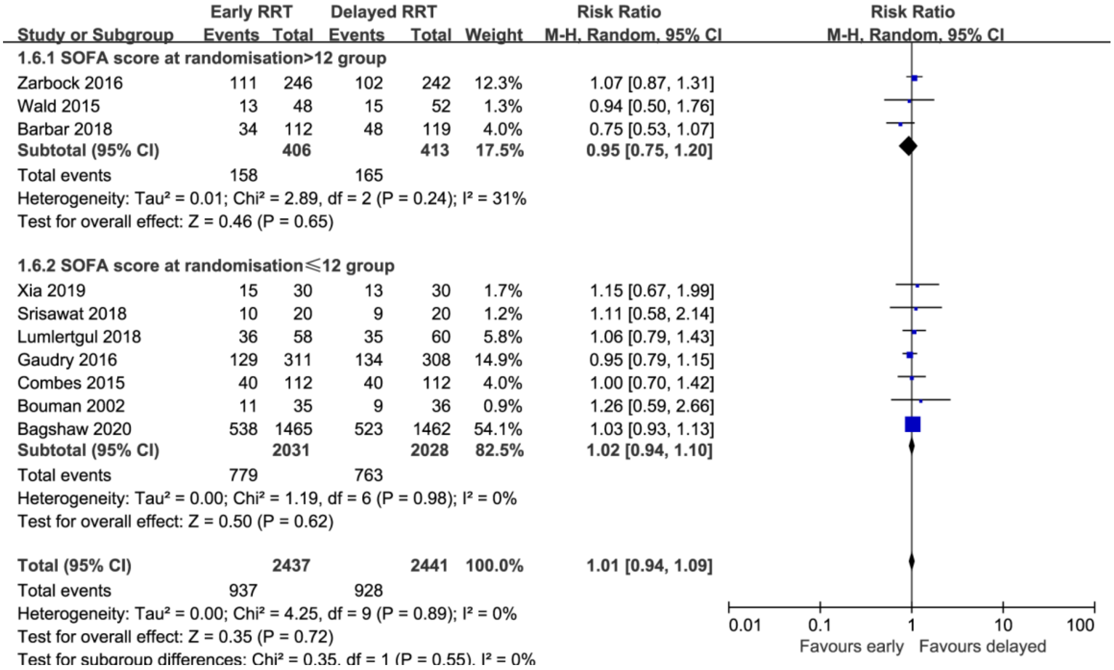

c. Subgroup analyses for 28-day mortality divided by the criteria for early RRT initiation

	Early RRT		Delayed RRT			Risk Ratio
Study or Subgroup	Events	Total	Events	Total	Weight	M-H, Fixed, 95% CI
**1.10.1 Approximately equal to stage 2 of the KDIGO classification**						
Bagshaw 2020	538	1465	523	1462	56.5%	1.03 [0.93, 1.13]
Bouman 2002	11	35	9	36	1.0%	1.26 [0.59, 2.66]
Wald 2015	13	48	15	52	1.6%	0.94 [0.50, 1.76]
Zarbock 2016	34	112	48	119	5.0%	0.75 [0.53, 1.07]
**Subtotal (95% CI)**		**1660**		**1669**	**64.0%**	**1.01 [0.92, 1.10]**
Total events	596		595			
Heterogeneity: Chi² = 3.12, df = 3 (P = 0.37); I² = 4%						
Test for overall effect: Z = 0.14 (P = 0.89)						
**1.10.2 Approximately equal to stage 3 of the KDIGO classification**						
Barbar 2018	111	246	102	242	11.1%	1.07 [0.87, 1.31]
Gaudry 2016	129	311	134	308	14.5%	0.95 [0.79, 1.15]
**Subtotal (95% CI)**		**557**		**550**	**25.6%**	**1.00 [0.88, 1.15]**
Total events	240		236			
Heterogeneity: Chi² = 0.69, df = 1 (P = 0.41); I² = 0%						
Test for overall effect: Z = 0.06 (P = 0.95)						
**1.10.3 Other classification criteria**						
Combes 2015	40	112	40	112	4.3%	1.00 [0.70, 1.42]
Lumlertgul 2018	36	58	35	60	3.7%	1.06 [0.79, 1.43]
Srisawat 2018	10	20	9	20	1.0%	1.11 [0.58, 2.14]
Xia 2019	15	30	13	30	1.4%	1.15 [0.67, 1.99]
**Subtotal (95% CI)**		**220**		**222**	**10.4%**	**1.05 [0.86, 1.29]**
Total events	101		97			
Heterogeneity: Chi² = 0.22, df = 3 (P = 0.97); I² = 0%						
Test for overall effect: Z = 0.51 (P = 0.61)						
**Total (95% CI)**		**2437**		**2441**	**100.0%**	**1.01 [0.94, 1.09]**
Total events	937		928			
Heterogeneity: Chi² = 4.25, df = 9 (P = 0.89); I² = 0%						
Test for overall effect: Z = 0.30 (P = 0.77)						
Test for subgroup differences: Chi² = 0.18, df = 2 (P = 0.91), I² = 0%						

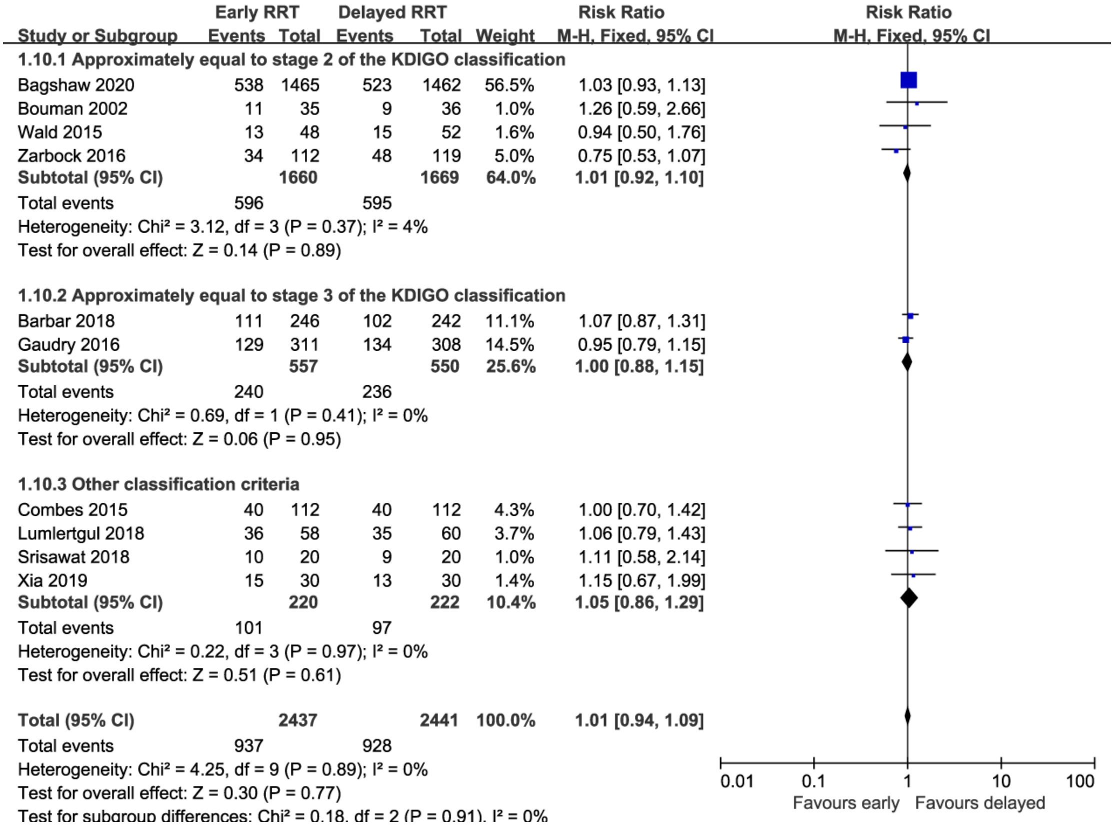

d. Sensitivity analyses according to publish language (excluding the study published in Chinese)

	Early RRT		Delayed RRT			Risk Ratio
Study or Subgroup	Events	Total	Events	Total	Weight	M-H, Fixed, 95% CI
Bagshaw 2020	538	1465	523	1462	57.3%	1.03 [0.93, 1.13]
Barbar 2018	111	246	102	242	11.2%	1.07 [0.87, 1.31]
Bouman 2002	11	35	9	36	1.0%	1.26 [0.59, 2.66]
Combes 2015	40	112	40	112	4.4%	1.00 [0.70, 1.42]
Gaudry 2016	129	311	134	308	14.7%	0.95 [0.79, 1.15]
Lumlertgul 2018	36	58	35	60	3.8%	1.06 [0.79, 1.43]
Srisawat 2018	10	20	9	20	1.0%	1.11 [0.58, 2.14]
Wald 2015	13	48	15	52	1.6%	0.94 [0.50, 1.76]
Zarbock 2016	34	112	48	119	5.1%	0.75 [0.53, 1.07]
**Total (95% CI)**		**2407**		**2411**	**100.0%**	**1.01 [0.94, 1.08]**
Total events	922		915			
Heterogeneity: Chi² = 4.03, df = 8 (P = 0.85); I² = 0%						
Test for overall effect: Z = 0.24 (P = 0.81)						

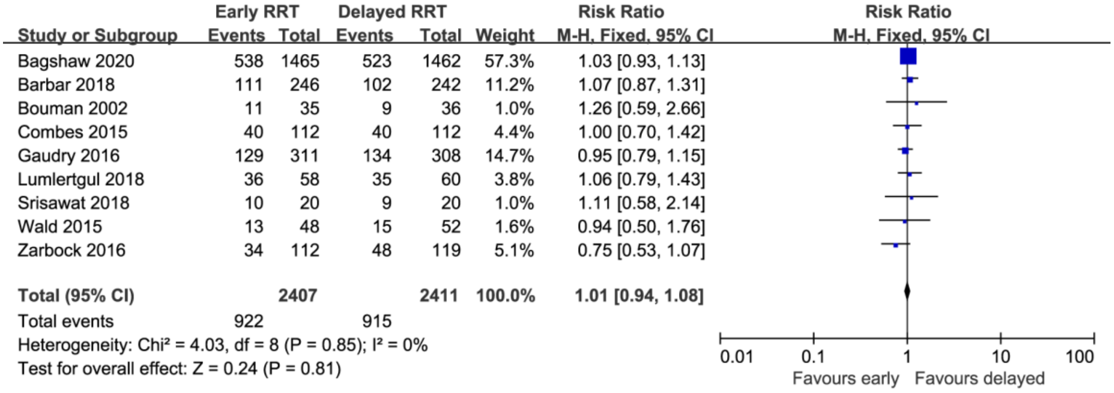

e. Sensitivity analyses according to risk of bias (only including studies classified as low risk of bias)

Study or Subgroup	Early RRT Events	Early RRT Total	Delayed RRT Events	Delayed RRT Total	Weight	Risk Ratio M-H, Fixed, 95% CI
Bagshaw 2020	538	1465	523	1462	57.8%	1.03 [0.93, 1.13]
Barbar 2018	111	246	102	242	11.4%	1.07 [0.87, 1.31]
Combes 2015	40	112	40	112	4.4%	1.00 [0.70, 1.42]
Gaudry 2016	129	311	134	308	14.9%	0.95 [0.79, 1.15]
Lumlertgul 2018	36	58	35	60	3.8%	1.06 [0.79, 1.43]
Srisawat 2018	10	20	9	20	1.0%	1.11 [0.58, 2.14]
Wald 2015	13	48	15	52	1.6%	0.94 [0.50, 1.76]
Zarbock 2016	34	112	48	119	5.1%	0.75 [0.53, 1.07]
**Total (95% CI)**		**2372**		**2375**	**100.0%**	**1.01 [0.94, 1.08]**
Total events	911		906			

Heterogeneity: Chi² = 3.70, df = 7 (P = 0.81); I² = 0%
Test for overall effect: Z = 0.17 (P = 0.86)

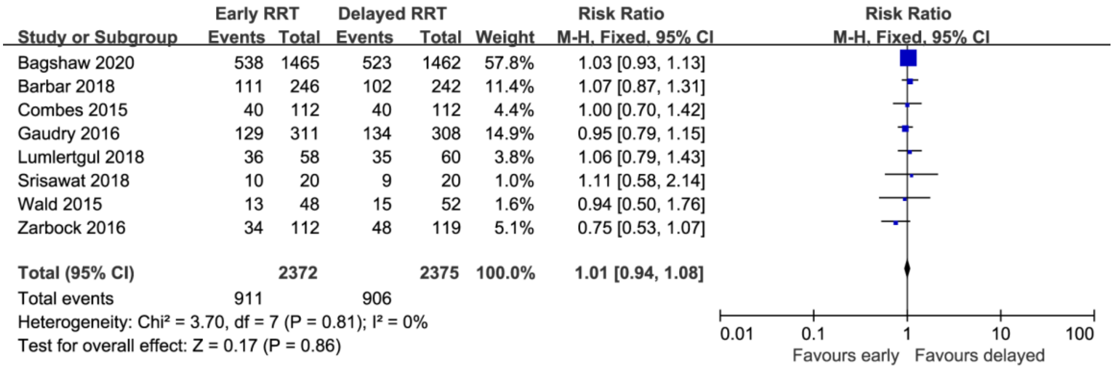

f. Sensitivity analyses according to publish year (removing studies published before 2010)

Study or Subgroup	Early RRT Events	Early RRT Total	Delayed RRT Events	Delayed RRT Total	Weight	Risk Ratio M-H, Fixed, 95% CI
Bagshaw 2020	538	1465	523	1462	57.0%	1.03 [0.93, 1.13]
Barbar 2018	111	246	102	242	11.2%	1.07 [0.87, 1.31]
Combes 2015	40	112	40	112	4.4%	1.00 [0.70, 1.42]
Gaudry 2016	129	311	134	308	14.7%	0.95 [0.79, 1.15]
Lumlertgul 2018	36	58	35	60	3.7%	1.06 [0.79, 1.43]
Srisawat 2018	10	20	9	20	1.0%	1.11 [0.58, 2.14]
Wald 2015	13	48	15	52	1.6%	0.94 [0.50, 1.76]
Xia 2019	15	30	13	30	1.4%	1.15 [0.67, 1.99]
Zarbock 2016	34	112	48	119	5.1%	0.75 [0.53, 1.07]
**Total (95% CI)**		**2402**		**2405**	**100.0%**	**1.01 [0.94, 1.08]**
Total events	926		919			

Heterogeneity: Chi² = 3.93, df = 8 (P = 0.86); I² = 0%
Test for overall effect: Z = 0.23 (P = 0.82)

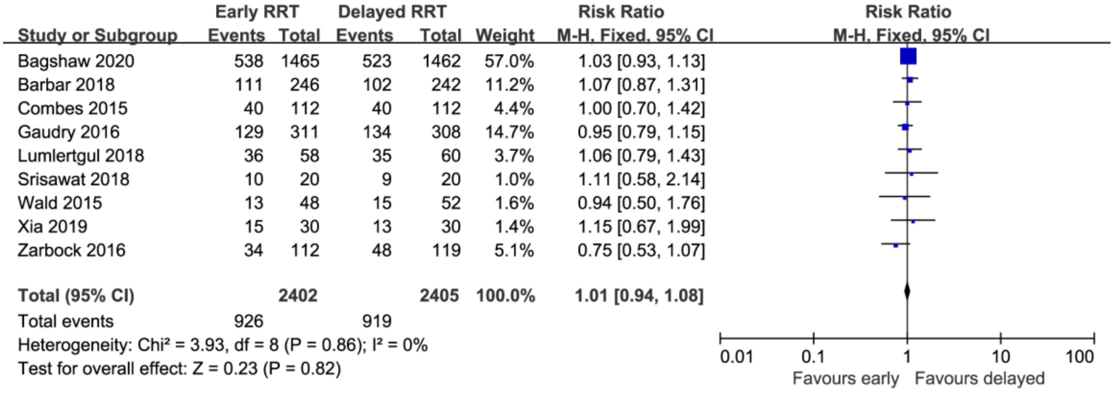

Supplement: Supplementary file 6 — Additional file 6: Forest plot of the subgroup analyses and sensitivity analyses: a.Subgroup analyses for 28-day mortality divided by the mean age of participants in each study; b. Subgroup analyses for 28-day mortality divided by the SOFA scores at administration; c. Subgroup analyses for 28-day mortality divided by the criteria for early RRT initiation; d. Sensitivity analyses according to publish language (excluding the study published in Chinese); e. Sensitivity analyses according to risk of bias (only including studies classified as low risk of bias); f. Sensitivity analyses according to publish year (removing studies published before 2010). [file 13054_2020_3451_MOESM6_ESM.pdf]
