# Supplementary material for: Timing of renal replacement therapy initiation for acute kidney injury in critically ill patients: a systematic review of randomized clinical trials with meta-analysis and trial sequential analysis
Source: Crit Care. 2021 Jan 6;25:15. doi: 10.1186/s13054-020-03451-y (PMC7789484; doi:10.1186/s13054-020-03451-y)
Supplement: Supplementary file 7 — Additional file 7: The inclusion criteria for each of the included studies in this meta-analysis. [file 13054_2020_3451_MOESM7_ESM.docx]

**The inclusion criteria for each of the included studies in this meta-analysis.**

| **Study** | **Inclusion criteria** |
| --- | --- |
| Bagshaw2020 | Patients were eligible if they were 18 years or older and had been admitted to an ICU with kidney dysfunction. |
| Barbar2018 | Patients were eligible if they were 18 years of age or older, were admitted to the ICU in the early phase of septic shock (within 48 hours after the start of vasopressor therapy), and had acute kidney injury for the failure stage of the RIFLE classification. |
| Bouman2002 | Ventilated severely ill ICU patients who were oliguric despite massive fluid resuscitation, inotropic support, and high-dose intravenous diuretics. |
| Combes2015 | Patients were eligible if they had undergone cardiac surgery and had persistent postoperative shock within 3–24 h following ICU admission.  unit (ICU) admission. |
| Gaudry2016 | Patients were eligible if they were adults (18 years of age or older) who were admitted to the ICU with acute kidney injury. |
| Jamale2013 | Participants were eligible for randomization if they had severe AKI with increasing serum urea nitrogen and creatinine levels. |
| Lumlertgul2018 | All adult patients (≥ 18 years old) admitted to the ICU were screened. |
| Srisawat2018 | ICU patients aged of 18 years or older who were diagnosed with AKI by RIFLE criteria.  diagnosed with AKI by RIFLE criteria |
| Wald2015 | Adults (≥18 years of age) admitted to an ICU with evidence of kidney dysfunction.  ≥130μmol/l for men).  dysfunction (defined as a serum creatinine ≥100μmol/l for women or  ≥130μmol/l for men).  age) admitted to an intensive care unit with evidence of kidney  dysfunction (defined as a serum creatinine ≥100μmol/l for women or  ≥130μmol/l for men). |
| Xia2019 | Patients who were diagnosed with sepsis AKI and with uNGAL greater than 1310 ng/ml. |
| Zarbock2016 | ICU patients with AKI Kidney Disease: Improving Global Outcomes (KDIGO) stage 2 and plasma neutrophil gelatinase–associated lipocalin level higher than 150 ng/mL were enrolled. |
